# Randomised controlled trial of a just-in-time adaptive intervention (JITAI) smoking cessation smartphone app: the Quit Sense feasibility trial protocol

**DOI:** 10.1136/bmjopen-2020-048204

**Published:** 2021-04-26

**Authors:** Felix Naughton, Chloë Brown, Juliet High, Caitlin Notley, Cecilia Mascolo, Tim Coleman, Garry Barton, Lee Shepstone, Stephen Sutton, A Toby Prevost, David Crane, Felix Greaves, Aimie Hope

**Affiliations:** 1 Behavioural and Implementation Science Group, School of Health Sciences, University of East Anglia, Norwich, UK; 2 Department of Computer Science and Technology, University of Cambridge, Cambridge, UK; 3 Norwich Clinical Trials Unit, University of East Anglia, Norwich, UK; 4 Norwich Medical School, University of East Anglia, Norwich, UK; 5 Division of General Practice, University of Nottingham, Nottingham, UK; 6 Behavioural Science Group, University of Cambridge, Cambridge, UK; 7 Nightingale-Saunders Clinical Trials & Epidemiology Unit, King's College London, London, UK; 8 Department of Behavioural Science and Health, University College London, London, UK; 9 Department of Primary Care and Public Health, Imperial College London, London, UK

**Keywords:** public health, preventive medicine, qualitative research

## Abstract

**Introduction:**

A lapse (any smoking) early in a smoking cessation attempt is strongly associated with reduced success. A substantial proportion of lapses are due to urges to smoke triggered by situational cues. Currently, no available interventions proactively respond to such cues in real time. Quit Sense is a theory-guided just-in-time adaptive intervention smartphone app that uses a learning tool and smartphone sensing to provide in-the-moment tailored support to help smokers manage cue-induced urges to smoke. The primary aim of this randomised controlled trial (RCT) is to assess the feasibility of delivering a definitive online efficacy trial of Quit Sense.

**Methods and analyses:**

A two-arm parallel-group RCT allocating smokers willing to make a quit attempt, recruited via online adverts, to usual care (referral to the NHS SmokeFree website) or usual care plus Quit Sense. Randomisation will be stratified by smoking rate (<16 vs ≥16 cigarettes/day) and socioeconomic status (low vs high). Recruitment, enrolment, baseline data collection, allocation and intervention delivery will be automated through the study website. Outcomes will be collected at 6 weeks and 6 months follow-up via the study website or telephone, and during app usage. The study aims to recruit 200 smokers to estimate key feasibility outcomes, the preliminary impact of Quit Sense and potential cost-effectiveness, in addition to gaining insights on user views of the app through qualitative interviews.

**Ethics and dissemination:**

Ethics approval has been granted by the Wales NHS Research Ethics Committee 7 (19/WA/0361). The findings will be disseminated to the public, the funders, relevant practice and policy representatives and other researchers.

**Trial registration number:**

ISRCTN12326962.

**Strength and limitations of this study**

- This is the first randomised controlled trial to evaluate a context-aware just-in-time adaptive intervention for smoking cessation.
- The study will identify key parameters to inform a definitive trial and will estimate intervention impact on biochemically validated abstinence from smoking.
- Recruitment, enrolment, baseline data collection, allocation, intervention delivery and most data collection during follow-up will be fully automated through the study website.
- There is potential for selection bias due to the requirement for participants to have internet access and an Android smartphone.

## Introduction

Tobacco smoking is the second largest contributor of disease burden globally^1^ and the largest in the UK.^2^ While quitting reduces the risk of smoking-related health problems and improves mental health,^3 4^ the success rate remains low. Of the three million UK smokers attempting to quit annually, over 80% relapse within 12 months.^5^

Almost half of all lapses (any smoking) during a quit attempt are attributed to cravings triggered by environmental or situational cues^6^ and lapses early in a quit attempt are highly predictive of longer-term relapse.^7–9^ However, the most commonly used cessation medications do not alleviate cue-induced cravings.^10 11^ Furthermore, while smokers are less likely to lapse if they use an effective cognitive and/or behavioural lapse prevention strategy,^10 12 13^ few do so.^12 14^ There is a need for cessation interventions that effectively support the management of cue-induced cravings.

Unlike traditional cessation interventions, smartphone apps have the advantage of portability and could therefore better assist with cue-induced cravings as they occur. Some mobile phone-based interventions have features specifically aimed at reducing lapse risk. Such support can be triggered by random Ecological Momentary Assessment prompts^15^ (where the smartphone prompts the user to complete assessment questions); or it can take the form of on-demand (ie, user-initiated) requests for craving support.^16 17^ However, given the rapid time to lapse after craving onset^10^ and that many on-demand craving tools are seldom used beyond a first try,^17 18^ these kinds of user-initiated features may still miss many craving episodes.

To address such limitations in current digital support, we developed Quit Sense, a digital intervention which predicts and adapts to smoking risk in real time^19^ (a just-in-time adaptive intervention). Quit Sense is a theory-guided smoking cessation tool that delivers ‘context-aware’ lapse prevention support primarily using smartphone location sensing. It has demonstrated feasibility in delivering ‘in the moment’ support and acceptability among users.^20^ We are now conducting a feasibility randomised controlled trial (RCT) of Quit Sense to inform the design parameters of a future RCT and to provide estimates of intervention impact.

This trial is being conducted during the COVID-19 pandemic and as such different contextual variables may impact on study participants during their quit attempts (eg, self-isolation, remote working) compared with before the COVID-19 pandemic. Through qualitative interviews and app recorded feedback, this feasibility trial will capture participant views of the acceptability of the app and study, including how these are impacted by any COVID-19 restrictions.

### Objectives

In this feasibility trial, we aim to estimate: (a) completion rates for the anticipated primary outcome for a full trial, (b) usual care arm cessation rate, (c) cost of recruitment using online advertising, (d) rates of app installation and use, and support delivery fidelity, (e) completion of smoking cessation-related resource use and quality of life (QoL) data, (f) participant views of the app, as part of a qualitative process evaluation, (g) intervention effect on anticipated primary outcome and (h) intervention effect on hypothesised mechanisms of action of app at 6 weeks postenrolment.

## Methods and analysis

### Study design

A two-arm parallel-group randomised controlled feasibility trial, allocating smokers to either ‘usual care’ (link to NHS SmokeFree website)^21^ or the intervention arm who receive ‘usual care’ plus access to the Quit Sense app.

Additionally, there is a nested qualitative process evaluation with a subsample of control and intervention participants to (1) assess intervention experiences and how these might help explain causal pathway(s) towards smoking behaviour change (intervention arm only)^22^ and (2) asses experiences of study participation.

Additional information on study configuration can be found in online supplemental appendix 1.

10.1136/bmjopen-2020-048204.supp1 Supplementary data

### Participants

#### Eligibility criteria

Participants will be smokers aged 16 years plus; currently smoke at least seven cigarettes per week; willing to make a quit attempt within 14 days of enrolling; have primary use of an Android smartphone (version 5.0 and above) and will be resident in England. Participants must not have previously participated in this trial.

### Study setting

Participants will complete screening, consent, baseline and follow-up measures online. This online design matches the ‘real world’ setting of smartphone app uptake.

### Online recruitment

**Figure 1:**
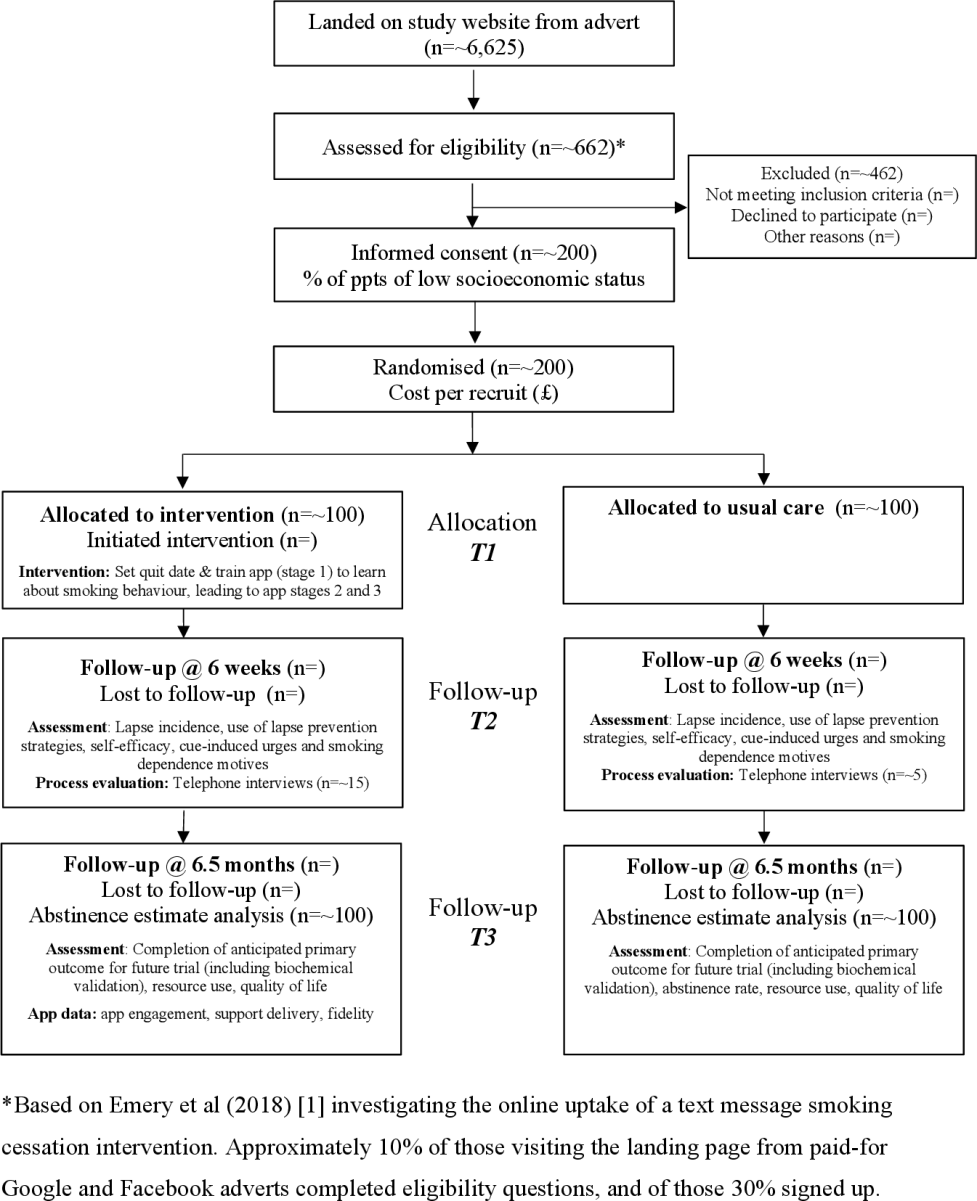
Flow diagram showing study design, measures and sample sizes.

Recruitment is through paid-for online advertisements with Google Search and social media (Facebook and Instagram), limited to England-based IP addresses and targeted at Android devices. Advertisements are managed by ‘Nativve’,^23^ specialists in digital marketing for research study recruitment. To maximise participation rate efficiency and in accordance with patient and public involvement (PPI) recommendations, different adverts will be tested using A/B experiments while recruitment is live. People clicking on adverts will be directed to the study website and provided with study information including a downloadable ‘Participant Information Sheet’. The screening and consent procedures are online with an e-signature confirming consent (table 1 and online supplemental appendix 2). After completing an online baseline questionnaire, participants will be randomly allocated to usual care or usual care plus Quit Sense. As described in figure 1 and informed by a prior text message smoking cessation intervention,^24^ we estimate that ~6625 unique visitors are required to achieve a sample size of 200.

10.1136/bmjopen-2020-048204.supp2 Supplementary data

**Table 1:** Schedule of participant assessments and intervention delivery

	Study period			
	Enrolment (online)	Allocation (online)	Postallocation (online or telephone)	Close-out (online or telephone)
Timepoint	0	0	*6 weeks FU (from enrolment)	†6 months FU (from enrolment)
Enrolment:				
Eligibility screen	X			
Informed consent	X			
Allocation		X		
Interventions:				
Usual care (link to www.smokefree.nhs.uk)		X		
Quit Sense app+usual care		X		
Assessments:				
Demographics	X			
Use of smartphone and apps	X			X
Smoking behaviour and dependence	X			X
Smoking beliefs	X			X
Cessation self-efficacy	X		X	X
Strength and frequency of urges to smoke	X		X	X
Automaticity and associative processes subscales of the WISDM-37	X		X	
EQ-5D-5L	X			X
Smoking lapse incidence			X	
Smoking abstinence outcomes			X	X
Use of lapse prevention strategies			X	
Smoking cessation resource use				X
Views on the app (Quit Sense arm only)				X
Assessment of tobacco exposure (postal saliva sample)				X

*Follow-up at 6 weeks (1 month, plus 2 weeks to cover the likely quitting period/phase 1 of the app).

†Follow-up will be scheduled approximately 6 months postenrolment in the study, allowing for an additional 2 weeks to cover the likely quitting period (phase 1 of the app). Although in practice this will be 6 months plus 2 weeks, we will refer to it simply as the ‘6-month follow-up’ for convenience.

EQ-5D-5L, quality of life; FU, follow-up; WISDM-37, Wisconsin Inventory of Smoking Dependence Motives.

The distribution of socioeconomic status (SES) during recruitment will be monitored. Low SES is defined as individuals with a semiroutine or routine and manual occupation, class 5 in the National Statistics Socio-Economic Classification (NS-SEC),^25^ or who have never worked or are long-term unemployed. Targeted advertising will be used to increase low SES representation if less than 45% of our sample are categorised as low SES when 35% of the target sample is reached.

### Procedure

Both arms will automatically receive a link to NHS SmokeFree via SMS after enrolment. Only the Quit Sense arm will receive an SMS with an app link and activation code. Use of the activation code will be monitored. Participants who do not initiate the app will be sent an SMS reminder 3 days postenrolment and then 5 days postenrolment asking to identify a reason for not initiating the app from a predefined list.

To attempt to increase response rates, all participants will receive an SMS prior to receiving links to follow-up questionnaires. Those who do not respond to the SMS with a survey link will be telephoned. Once the 6-week questionnaire is completed, a subsample of participants from both arms (approximately 3:1 ratio between Quit Sense and usual care) will be invited to a telephone interview to gather data on their experiences of the app (intervention only) and of participating in the study. Where practicable, we will use purposive sampling to interview a mixture of high and low SES abstainers and smokers, and for Quit Sense participants based on varying rates of app engagement. We anticipate interviewing 20 participants, depending on when data saturation is reached.

Six months and 2 weeks after enrolling (allowing for a 2-week quitting preparation period for Quit Sense participants; henceforth, referred to as the ‘6-month’ follow-up), all participants will be asked via SMS to complete the final questionnaire. Participants reporting abstinence in the previous 7 days at follow-up will be posted a saliva sample kit to be returned to ABS laboratories, UK.

Participants will receive vouchers on completion of the 6-week (£5) and 6-month (£10) surveys, return of the saliva sample (£5) and for participating in an interview (£20).

As suggested by our PPI panel, all participants will receive a postcard and SMS to thank them and emphasise their importance to the study.

### Criteria for discontinuing or modifying allocated interventions

Unless a participant withdraws from the study, they will be followed-up for data collection regardless of their level of engagement or smoking status. Participants, who discontinue using the app for any reason other than withdrawal, will remain in the trial for the purpose of follow-up and data analysis.

### Interventions

#### Usual care arm

Usual care is a web-link (NHS SmokeFree) sent by SMS. We consider this equivalent to ‘usual care’ for an online population. SmokeFree provides access and signposting to digital, telephone and in-person cessation support in England. At the time of writing, the support offered includes information and links to six main types of support; support from a cessation advisor (via a Stop Smoking Service), the SmokeFree National telephone helpline, an online chat facility with an advisor, an email cessation programme, an SMS text message programme and the NHS SmokeFree smartphone app (28-day programme, non-tailored and not including proactive support delivery for managing urges to smoke).

### Quit sense APP arm

Quit Sense is a context-aware smartphone app designed for smokers wanting to quit. It is informed by learning theory (LT)^26 27^ and two theory-guided SMS text message systems,^17 28^ which are in turn informed by social cognitive theory (SCT).^29^ Quit Sense targets three key determinants that map onto LT and six mapping onto SCT: learnt associations between smoking and an individual’s physical environment (LT); learnt associations between smoking and mood triggered by the environment (LT); the presence of other smokers (LT, SCT); awareness of smoking triggers (antecedents) (SCT); outcome expectancies of a lapse (SCT); goal/intention for complete abstinence (SCT); self-efficacy in resisting urges to smoke (SCT); knowledge of and self-efficacy for lapse prevention strategy use (SCT). Twenty-one corresponding behaviour change techniques (BCTs)^30^ are used to target these determinants.

The main feature is ‘Geofence-Triggered Support’ (GTS), which is orientated around three app stages:

- Stage 1 (training): the user sets a quit date (default suggestion 7 days) and trains the app to learn about their smoking behaviour. This requires the user to report in the app each smoking episode and the situational context in real time (stress, mood, urge strength, setting description (home, working, home-working, socialising, other), presence of other smokers), while the app records geolocation. If a user reports smoking more than once in the same location (as defined by the app), a geofence (a circular virtual perimeter) is created representing a high-risk area. Location monitoring precision varies depending on the availability of GPS/WiFi/network (range ~5–50 m).
- Stage 2 (28-day abstinence challenge): postquit date, the app monitors the user’s location. A support message is triggered a minimum of 1 min after the user enters a geofence zone (typical range 1–15 min, depending on operating system). Messages are individually tailored using stage 1 context information and using any stage 2 smoking reports, providing support to help users avoid or cope with location specific triggers. Further messages are triggered after each 3-hour interval of remaining in that location (default between 8 am and 9.30 pm or as defined by the user). This also applies to ‘home-working’ messages. However, if the user has not reported ‘home-working’ within the time window (2–3 hours) in which the message is to be delivered, a ‘home’ message will be delivered instead. This is because ‘time’ of reporting is an additional requirement (to ‘location’) for home-working messages to be triggered.
- Stage 3 (maintaining abstinence): the app continues to deliver GTS for two further months but reduces the frequency by one-half every month. After 3 months postquit date, the GTS support stops, unless the user opts to switch it back on or restart their quit attempt.

Quit Sense has six additional features (relevant to all stages, unless specified otherwise):

1. Feedback is provided after each smoking report. In stage 1 this is tailored to nine characteristics collected during app initiation. Messages focus on enhancing quitting preparation, motivation and self-efficacy. In stage 2, feedback is non-tailored and orientated around lapse and relapse prevention.
2. An ‘End of Day’ (EoD) survey that users are prompted to complete, recording cigarettes smoked that day, cravings^31^ and self-efficacy^28 29^ (stages 1 and 2 only). Feedback is provided after each EoD survey, including encouragement to log smoking behaviour if there is a discrepancy between the survey and real time reports.
3. A ‘my profile’ screen including number of days quit, money saved (based on smoking rate and reported cost), a calendar showing a heat map and ‘smileys’ (use of yellow, grey and blue to denote values with corresponding happy, neutral and sad smileys) for smoking, cravings and self-efficacy for each EoD survey completed. In addition, smoking pattern feedback is provided using graphical and written summaries for smoking triggers based on the user’s historical reporting.
4. A ‘library’ of quitting advice split into six categories: ‘getting ready’, ‘boost your motivation’, effects of smoking and quitting’, ‘early days of quitting’, ‘staying quit’, ‘smoking after your quit date’. Users can write and submit their own support messages.
5. Scheduled non-tailored daily support messages delivered each morning orientated around the quit date (stages 1 and 2 only)—targeting outcome expectancies, motivation, preparation, self-efficacy (three sets of different messages to prevent message duplication in successive attempts).
6. The option for the user to reset their quit date in stage 1, if they are not feeling ready, or stage 2 if they relapse. Relapse is defined as multiple smoking episodes that are reported over 2 days during a quit attempt (either through smoking episode logs or the EoD survey). If this occurs, users are invited to either recommit and continue or reset their quit date and return to Stage 1.

### Qualitative process evaluation

The aims of the qualitative process evaluation are:

1. To gather user experiences of app usage in order to inform app optimisation.
2. To understand participant experiences of participation and the unique contexts in which participants are attempting to quit.

Interview questions will probe for in-depth descriptions of intervention participants’ experiences of app usage including which types of support they liked most and least, the timing of support in relation to experienced need for support, and views on the features used. Control participants will be asked about their experience of trial participation. Both groups will be asked about their use of or interest in other smoking cessation aids and support and contextual details such as mandated movement restrictions and family setting. In addition, the Quit Sense app includes an in-built audio record feature so that users can leave feedback. The study will therefore also assess the feasibility of a user-initiated process evaluation approach where data are collected with high ecologically validity. Any relevant data submitted will be analysed alongside interview data, potentially providing a broader range of views from a more user-controlled perspective. Transcriptions from interviews or app recordings will be anonymised. Recordings will be deleted after transcription, or if not fully transcribed, after audio coding for analysis.

### Database

Study data are collected and managed using REDcap (Research Electronic Data Capture) tools hosted at Norwich Clinical Trials Unit. REDcap is a secure, web-based software platform supporting data capture for research studies.^32 33^ Further details on data management can be found in the online Supplementary File.

### Outcomes

#### Feasibility outcomes

As this is a feasibility trial, there is no primary outcome. Feasibility outcomes will be used to estimate key parameters to inform a future trial,^34^ and include:

1. Completeness of the anticipated primary outcome for a future definitive trial (6-month self-reported abstinence with biochemical validation; see smoking outcomes section).
2. Abstinence rate of usual care arm, using the anticipated primary outcome for a future definitive trial.
3. Cost per recruit, based on advertising costs.
4. Rates of app installation use and support delivery fidelity.
5. Completion of smoking cessation-related resource use, including usual care use and quality of life (EQ-5D-5L)^35^ data.
6. Hypothesised mechanisms of action of Quit Sense.
7. Participant experiences and feedback on app usage.

### Smoking and related outcomes

Smoking and related outcomes will provide preliminary information about the intervention’s impact. The main abstinence outcome for which we will monitor completeness of ascertainment is based on the Russell standard.^36^ Abstinence will be defined as self-reported abstinence in the previous 6 months allowing for no more than five cigarettes and not smoking in the previous week, biochemically validated by a saliva cotinine concentration of less than 10 ng/mL^36 37^ and for those using nicotine substitution (eg, Nicotine Replacement Therapy, or e-cigarettes) an anabasine concentration of less than 0.2 ng/mL, based on feedback from our testing laboratory.^37 38^ Thresholds will be reviewed prior to analysis in case of changes in guidance or relevant evidence. Given recent evidence that some e-liquid, believed to be that made outside of the UK, can contain anabasine,^37^ we will monitor the proportion of e-cigarette users who report abstinence from smoking but have an anabasine level above the chosen threshold.

Smoking cessation experts highlight the value of additional smoking outcomes based on different time periods of abstinence and time-points.^39^ We will therefore measure 7-day point prevalence abstinence at 6 months follow-up (self-report and biochemically verified) and 7-day point prevalence abstinence at 6 weeks follow-up (self-report).

We will collect the following hypothesised mechanisms of action at 6-week follow-up:

- Lapse incidence (any smoking, even a puff) since the initial quit attempt (if made),^6^ which is highly predictive of relapse.^7–9^
- Lapse prevention strategy use,^14^ which is associated with lapse prevention.^10^
- Self-efficacy,^28 29^ which prospectively predicts lapse and relapse to smoking.^40^
- The strength of urges to smoke measure,^31^ which prospectively predicts abstinence and is superior to other urge-measures in doing so,^41^ and frequency of urges to smoke.^41^
- Automaticity and associative processes subscales from the Wisconsin Inventory of Smoking Dependence Motives (WISDM-37).^42^

### Sample size

In line with recommendations that feasibility studies with binary outcome measures recruit at least 60 participants per group (minimum of 120) and a maximum of 100 per group, the proposed sample size for this trial is 200 smokers (100 per arm).^43^ Participants will be randomised to usual care or Quit Sense arms on a 1:1 ratio. This sample size will enable key parameters to inform a future definitive trial to be estimable within the following precision (defined as the 95% CI half-width):

- Primary outcome completion: we estimate a follow-up rate for self-reported smoking status at 6 months of 80%, with precision of ±6%.^44–46^ For biochemical validation, we estimate that 75% of participants reporting abstinence will return a saliva sample by post, with precision of ±22%.^28 45^
- Cessation rate in usual care arm: we estimate an abstinence rate of 5% at 6 months follow-up, providing precision of±4%.^47^
- App installation and initiation: we estimate that 85% of intervention participants will instal the app, with precision of±7%.^48^
- App engagement—in our acceptability study, 71% used the app for more than 1 week (a timeframe deemed meaningful by our PPI panel). With a similar rate in the trial, precision would be ±9%. This is in line with other studies.^48^

### Randomisation

#### Sequence generation, allocation and blinding

Randomisation will be stratified by smoking rate (<16 vs ≥16 cigarettes/day; based on mean smoking rates from trials recruiting smokers online)^45 49^ and SES (low vs high), based on the NS-SEC self-coded method.^50^ Allocation sequences will be generated by a computer-based random number generator using random permuted blocks (varying block sizes) (using REDcap). Randomisation will be integrated into the enrolment process on the study website. The system will not permit a participant to enrol twice with the same mobile number or email address. As allocation will be integrated into the study website, the allocation sequence will be concealed from participants until assignment and concealed from members of the trial team, except the statistician, developers of the study database and the research associate supporting trial delivery.

### Data analysis

The feasibility and acceptability of all measures will be assessed by their level of completeness and via interviews and app audio data.

### Outcomes

Completeness of the anticipated primary outcome for a future trial (outcome i) and the abstinence rate using this anticipated primary outcome for the usual care arm (outcome ii) will be described as proportions with 95% CIs, and translated into interpretable probabilities using the Bayesian approach relevant in preliminary trials with the objective of powering the definitive trial.

Cost per recruit (outcome iii), rates of app installation (outcome iv) and completion of smoking cessation-related resource use and EQ-5D-5L (outcome v) will be described using summary statistics with 95% CIs.

We will estimate the preliminary intervention effect on abstinence, lapse incidence and use of lapse prevention strategies, using multiple logistic regression, providing ORs with 95% CIs, while adjusting for any potential baseline confounders defined by the statistical analysis plan (SAP). For the estimated intervention effect on abstinence, we will assume missing=smoking.^36^ To estimate intervention effects on self-efficacy, urges and smoking dependence motives, we will use analysis of covariance models involving adjustment for the baseline score and for predefined potential confounders, primarily using complete cases only. Missing outcome assumptions will be assessed through sensitivity analyses which will be defined in the SAP.

### Economic evaluation

We aim to collect data (baseline and 6-months follow-up) that will enable us to inform the decision as to how cost (based on resource use data) and benefit (in terms of QoL) data are estimated in any future more definitive study. Completion rates will thereby be estimated.

In terms of costs, we will estimate those associated with the intervention (eg, app maintenance) and smoking related costs to both the individual (eg, nicotine replacement therapy) and the NHS (eg, NHS stop smoking services/GP visits). In line with National Institute for Health and Care Excellence (NICE) guidance,^51^ QoL will be measured via the EQ-5D-5L^35^ as this enables the calculation of QALY (Quality Adjusted Life Year) scores. Appropriate unit costs^52^ will also be attached to all items of resource-use in order to identify major cost drivers.

Based on the intention to treat approach, a preliminary cost-effectiveness analysis will be performed. In the base-case analysis, costs will be estimated from the viewpoint of the NHS and personal social services.^51^ We will estimate the mean incremental cost and mean QALY gain associated with Quit Sense compared with usual care. Assuming dominance does not occur (where one intervention is both more costly and less effective), then the incremental cost effectiveness ratio (incremental cost/QALY gain) will be estimated and assessed in relation to a range of cost-effectiveness thresholds (eg, £20 000 to £30 000 per QALY),^51^ in order to provide a preliminary assessment of whether Quit Sense constitutes value for money. The associated level of uncertainty will also be estimated.

### Qualitative process evaluation data collection and analysis

Interviews will be audio-recorded and transcribed intelligent verbatim. App audio-recordings may be transcribed depending on quality and resources. If transcription is unfeasible, coding of audio files will be completed.

We will undertake an inductive thematic analysis of the first 3–4 interview transcripts,^53^ develop a coding framework from this and then code remaining data. We will check coding consistency through the independent coding of 10% of the data, with a further 10% if consistency is unsatisfactory. We will continue refining until the themes are agreed.

Analysis of process evaluation data will focus on identifying key themes associated with using the app and views and experiences of using other relevant apps. A second stage of analysis will work with descriptions of experiences of lapse or lapse avoidance in the context of the app and considering unique participant contexts. These will be reported as vignettes and used to explore participant experiences and to identify mechanisms of action and casual pathways towards behaviour change.

### Patient and public involvement

We have a PPI panel of four former smokers who have contributed to study design including the recruitment strategy, wording of SMS messages, study information, the qualitative evaluation, the design of the study logo and the function and features of the Quit Sense app and support it delivers. The PPI panel is represented on the Trial Steering Committee.

## Discussion

A feasibility trial of Quit Sense will identify the conditions necessary for a definitive trial, including for successful study design and data collection. This will largely be determined by whether the recruitment strategy is able to produce the required sample size and participant retention rates; the rate of app installation; completion rates for follow-up and the anticipated primary outcome for a full trial and participant views of the acceptability of the app and study. A potential future RCT will enable us to establish the extent to which the Quit Sense app is effective and cost effective relative to the usual provision of online stop smoking support.

This remotely delivered and largely automated trial is being conducted during the COVID-19 pandemic, a time when health protection measures are restricting or preventing many everyday behaviours. As such, there are likely to be different contextual variables impacting on study participants (eg, self-isolation, remote working, limited opportunities to socialise, etc.) compared with before the pandemic. The Quit Sense app can learn about such situational factors because of the initial training phase where the user reports on their smoking behaviour. Furthermore, we hope to capture in-depth insights into the unique contexts in which participants are attempting to make their quit attempts through telephone interviews and the app’s inbuilt audio-record feature.

The potential of intelligent and ‘always on’ digital cessation tools in providing on-hand effective and low cost support means that they are likely to be of growing importance in enhancing chances of successful quitting.^54^ However, measures are needed to realise the potential of next generation digital cessation interventions most of which remain untested.^15^ Where app evaluations have been undertaken results show that evidence-based smoking cessation strategies are underutilised,^55^ that many use a limited number of BCT,^56 57^ and that few use features such as geolocation or just-in-time sensors.^56^ It is crucial, therefore, that their effectiveness and cost effectiveness are established, particularly through use of randomised control trials.^58^

Quit Sense has been developed to address many of the current limitations in digital support. The app is distinct from other cessation support available due to its ability to identify location-based high-risk locations using smartphone sensing to provide timely theory-guided and tailored ‘in-the-moment’ support. Quit Sense has been developed to help smokers avoid or manage urges to smoke triggered by cues within these high-risk locations. This is done through the provision of lapse prevention strategies, outcome expectancy management and motivational and self-efficacy enhancement. Ultimately, this support aims to help extinguish learnt associations between smoking and a person’s physical, social and psychological environment in order to reduce future relapse risk.

### Ethics and dissemination

Ethics approval has been granted by the Wales NHS Research Ethics Committee 7 (19/WA/0361). The findings will be disseminated to the public, the funders, relevant practice and policy representatives and other researchers. A fully anonymised data set from the trial will be made available on reasonable request.

## Supplementary Material

Reviewer comments

Author's manuscript
