# Supplementary material for: Randomised controlled trial of a just-in-time adaptive intervention (JITAI) smoking cessation smartphone app: the Quit Sense feasibility trial protocol
Source: BMJ Open. 2021 Apr 26;11(4):e048204. doi: 10.1136/bmjopen-2020-048204 (PMC8076923; doi:10.1136/bmjopen-2020-048204)
Supplement: Supplementary data [file bmjopen-2020-048204supp001.pdf]

Supplementary File

Protocol amendments.

Minor, non-notifiable, August 2020.

Minor, non-notifiable, December 2020.

Amendments to the Protocol and other documents (e.g. changes to eligibility criteria, outcomes, sample size calculations, analyses) will be agreed by the Trial Management Group (TMG). Such amendments will be forwarded to the Sponsor for confirmation as to whether it is either substantial or non-substantial and will then be submitted to the Ethics Committee for categorisation and approval prior to implementation.

Indemnity

University of East Anglia insurance shall apply.

Ancillary and post-trial care

The end of the trial is defined as 6 months following the last follow-up of the last participant randomised, to allow for data entry and data cleaning activities to be completed.

Once participants' involvement in the study has ended, they will no longer receive communications from the study team other than a copy of the findings, if they have requested one. Participants will be sent an SMS to inform them that their participation in the study has ended.

The NHS SmokeFree website support will continue be available after this study ends. Participants in the app group will still be able to access and use the Quit Sense app on their mobile if they wish to do so. If participants continue to use the app, it will continue to collect research data that we may use as part of the study analyses. After the study ends Quit Sense will no longer have technical support and participants will not be able to update or reinstall it. If participants wish, they can uninstall the app following the usual process for removing apps from their phone.

Naughton F, *et al. BMJ Open* 2021; 11:e048204. doi: 10.1136/bmjopen-2020-048204

Data

Monitoring and management

The Chief Investigator has overall responsibility and will oversee study management. The Trial Management Group will be responsible for the running of the trial and will be supported by the Trial Steering Committee. A separate Data Monitoring and Ethics Committee is not considered necessary because the study is deemed to be low risk to participants.

Study data are collected and managed using REDcap (Research Electronic Data Capture). Data provided by trial participants will be entered under the participants PID number onto the central database stored on the servers based at the Norwich Clinical Trials Unit (NCTU). Access to the database will be via unique, individually assigned (i.e. not generic) usernames and passwords, and only accessible to members of the Quit Sense trial team and external regulators if requested. The servers are protected by firewalls and are patched and maintained according to best practice. The physical location of the servers is protected physically and environmentally in accordance with University of East Anglia's General Information Security Policy 3 (GISP3: Physical and environmental security).

The database and associated code have been developed by NCTU Data Management in conjunction with the Quit Sense trial team. The database software provides several features to help maintain data quality, including; maintaining an audit trail, allowing custom validations on all data, allowing users to raise data query requests, and search facilities to identify validation failure/ missing data. After completion of the trial the database will be retained on the servers of NCTU for on-going analysis of secondary outcomes.

Any transcriptions from the telephone interviews or app collected qualitative data will be anonymised. Voice recordings from telephone interviews and app recordings will be deleted after transcription, or if not transcribed then after audio coding for analysis.

Confidentiality and access to data

The investigators agree to archive and/or arrange for secure storage of Quit Sense trial materials and records for 20 years after the close of the trial unless otherwise advised by the NCTU. Personal contact details such as names, addresses and phone numbers will be destroyed 12 months after the end of the trial. Requests for access to trial data will be

considered, and approved in writing where appropriate, after formal application to the TMG or Trial Steering Committee (TSC). Considerations for approving access are documented in the TMG and TSC Terms of Reference. Location data will be kept for 20 years before being destroyed. This data will not be made available as an open data set outside of Quit Sense collaborations.

Concomitant Care

All participants will have access to treatment as usual for smoking cessation regardless of randomisation into this trial.

Roles and responsibilities

These membership lists are correct at the time of writing:

Protocol contributors

Name	Affiliation	Role
Felix Naughton	UEA	Led on protocol development
Juliet High	Norwich CTU	Contributed to regulatory and CTU related content
Aimie Hope	UEA	Contributed to protocol development
Caitlin Notley	UEA	Contributed to the qualitative process evaluation section
Lee Shepstone	Norwich CTU	Contributed to the statistical analysis section
Garry Barton	Norwich CTU	Contributed to the health economics section
Tim Coleman	University of Nottingham	Detailed comments on draft

Role of trial sponsor and funders

Name	Affiliation	Role
Helen Sutherland	South Norfolk CCG (‘Host’)	Representative of the host organisation
Graham Horne	UEA (‘Sponsor’)	Representative of the sponsor
Sheila Turner	NIHR	Representative of the funder

Naughton F, *et al. BMJ Open* 2021; 11:e048204. doi: 10.1136/bmjopen-2020-048204

Trial Team

Name	Affiliation	Role and responsibilities
Felix Naughton	UEA	Chief investigator
Juliet High	Norwich CTU	Coordination of CTU contribution to project
Aimie Hope	UEA	Senior Research Associate; supporting trial delivery
Chloe Brown	University of Cambridge	Computer scientist leading app optimisation
Claire West	Norwich CTU	Development of study database and web-platform

Trial Management Group

Name	Affiliation	Role and responsibilities
Felix Naughton	UEA	Chief investigator
Juliet High	Norwich CTU	Coordination of CTU contribution to project
Aimie Hope	UEA	Senior Research Associate; supporting trial delivery
Tim Coleman	UEA	Co-applicant; mentoring support for CI
Caitlin Notley	UEA	Co-applicant; qualitative process evaluation advisor
Garry Barton	Norwich CTU	Co-applicant; health economics oversight
Chloe Brown	University of Cambridge	Computer scientist leading app optimisation
Ann Marie Swart	Norwich CTU	CTU oversight
Claire West	Norwich CTU	Development of study database and web-platform
Graham Horne	UEA	Representative of trial sponsor

Trial Steering Committee

Name	Affiliation	Role and responsibilities
Michael Ussher	St Georges/University of Stirling	Independent chair (clinical/behavioural science)
Aleksandra Herbec	UCL	Independent member (behavioural science)

Simon Coulton	University of Kent	Independent member (statistics/HE)
Qasim Chowdary	Public Health England	Independent member (services/clinical/policy)
Jo Hardy	PPI	Independent member (PPI)
Felix Naughton	UEA	Non-independent member (voting)
Juliet High	Norwich CTU	Observer
Helen Sutherland	South Norfolk CCG	Observer (host organisation)
Graham Horne	UEA	Observer (sponsor)
Aimie Hope	UEA	Observer

Naughton F, *et al. BMJ Open* 2021; 11:e048204. doi: 10.1136/bmjopen-2020-048204
