# Supplementary material for: Randomised controlled trial of a just-in-time adaptive intervention (JITAI) smoking cessation smartphone app: the Quit Sense feasibility trial protocol
Source: BMJ Open. 2021 Apr 26;11(4):e048204. doi: 10.1136/bmjopen-2020-048204 (PMC8076923; doi:10.1136/bmjopen-2020-048204)
Supplement: Supplementary data [file bmjopen-2020-048204supp002.pdf]

Appendix 2

Quit Sense Consent form

I understand that my participation is voluntary and that I am free to withdraw at any time, without giving any reason, and without my legal rights being affected.	
I understand that should I withdraw then the information collected so far cannot be erased and that this information may still be used in the project analysis.	
I understand that my personal details will be kept confidential.	
I understand that the data collected about me will be anonymised so that I am not identifiable, that study findings may be disseminated (e.g., in publications, academic conferences), and that fully anonymised data may be shared on academic open access platforms to ensure transparency, aid peer review and allow others to make use of important data for re-analysis.	
I understand and agree that I **may** be invited to verify my smoking status at the end of the study by giving a saliva sample by post, and that giving this sample is optional.	
I understand and agree that I **may** be invited to provide feedback on my experiences of this study and the digital support provided in a recorded telephone interview or by leaving a recorded message, and that giving my feedback is optional.	
I understand that if I provide feedback I may be quoted in published academic journals or presentations at public and academic events in the future, but to maintain my confidentiality I will not be named.	
I agree to my postal address being used in this study and consent to it being passed to the Quit Sense team. I understand my postal address will only be used for this study.	
I understand that **if** I am in the quit smoking app group, I will be offered access to a quit smoking app which will use my phone's in-built sensors (e.g. GPS) to record my location.	
I agree to my mobile phone number being used in this study and consent to it being passed to the Quit Sense team in case I will receive study related text messages. I understand my mobile number will only be used for this study.	
I agree to take part in the above study.	✓
Optional<br>Send me the findings.	✓

I agree to my contact details being held on a secure database until the end of the study so that I may receive a copy of<br>the results.
Name of participant<br><br>Date (entered automatically)
