# Supplementary material for: Randomised controlled trial of a just-in-time adaptive intervention (JITAI) smoking cessation smartphone app: the Quit Sense feasibility trial protocol
Source: BMJ Open. 2021 Apr 26;11(4):e048204. doi: 10.1136/bmjopen-2020-048204 (PMC8076923; doi:10.1136/bmjopen-2020-048204)
Supplement: Author's manuscript [file bmjopen-2020-048204.draft_revisions.pdf]

BMJ Open is committed to open peer review. As part of this commitment we make the peer review history of every article we publish publicly available.

When an article is published we post the peer reviewers' comments and the authors' responses online. We also post the versions of the paper that were used during peer review. These are the versions that the peer review comments apply to.

The versions of the paper that follow are the versions that were submitted during the peer review process. They are not the versions of record or the final published versions. They should not be cited or distributed as the published version of this manuscript.

BMJ Open is an open access journal and the full, final, typeset and author-corrected version of record of the manuscript is available on our site with no access controls, subscription charges or pay-per-view fees (http://bmjopen.bmj.com).

If you have any questions on BMJ Open's open peer review process please email info.bmjopen@bmj.com

BMJ Open

Randomised controlled trial of a Just-In-Time Adaptive Intervention (JITAI) smoking cessation smartphone app: The Quit Sense feasibility trial

Journal:	*BMJ Open*
Manuscript ID	bmjopen-2020-048204
Article Type:	Protocol
Date Submitted by the Author:	22-Dec-2020

Complete List of Authors:	Naughton, Felix; University of East Anglia, Behavioural and Implementation Science Group, School of Health Sciences<br>Brown, Chloë; University of Cambridge, Department of Computer Science and Technology<br>High, Juliet; University of East Anglia, Norwich Clinical Trials Unit<br>Notley, Caitlin; University of East Anglia, Norwich Medical School<br>Mascolo, Cecilia; University of Cambridge, Department of Computer Science and Technology<br>Coleman, Tim; University of Nottingham, Division of General Practice<br>Barton, Garry; University of East Anglia, Norwich Medical School<br>Shepstone, Lee; University of East Anglia, Norwich Clinical Trials Unit<br>Sutton, Stephen; University of Cambridge, Behavioural Science Group<br>Prevost, A.; King's College London, Nightingale-Saunders Clinical Trials & Epidemiology Unit<br>Crane, David; University College London, Department of Behavioural Science and Health<br>Greaves, Felix; Imperial College London, Department of Primary Care and Public Health<br>Hope, Aimie; University of East Anglia, Behavioural and Implementation Science Group, School of Health Sciences

Keywords:	PUBLIC HEALTH, PREVENTIVE MEDICINE, QUALITATIVE RESEARCH

SCHOLARONE™
Manuscripts

I, the Submitting Author has the right to grant and does grant on behalf of all authors of the Work (as defined in the below author licence), an exclusive licence and/or a non-exclusive licence for contributions from authors who are: i) UK Crown employees; ii) where BMJ has agreed a CC-BY licence shall apply, and/or iii) in accordance with the terms applicable for US Federal Government officers or employees acting as part of their official duties; on a worldwide, perpetual, irrevocable, royalty-free basis to BMJ Publishing Group Ltd ("BMJ") its licensees and where the relevant Journal is co-owned by BMJ to the co-owners of the Journal, to publish the Work in this journal and any other BMJ products and to exploit all rights, as set out in our licence.

The Submitting Author accepts and understands that any supply made under these terms is made by BMJ to the Submitting Author unless you are acting as an employee on behalf of your employer or a postgraduate student of an affiliated institution which is paying any applicable article publishing charge ("APC") for Open Access articles. Where the Submitting Author wishes to make the Work available on an Open Access basis (and intends to pay the relevant APC), the terms of reuse of such Open Access shall be governed by a Creative Commons licence – details of these licences and which Creative Commons licence will apply to this Work are set out in our licence referred to above.

Other than as permitted in any relevant BMJ Author's Self Archiving Policies, I confirm this Work has not been accepted for publication elsewhere, is not being considered for publication elsewhere and does not duplicate material already published. I confirm all authors consent to publication of this Work and authorise the granting of this licence.

For peer review only

Title: Randomised controlled trial of a Just-In-Time Adaptive Intervention (JITAI) smoking cessation smartphone app: The Quit Sense feasibility trial

Short title: Quit Sense feasibility trial

Felix Naughton[1], Chloë Brown[2], Juliet High[3], Caitlin Notley[4], Cecilia Mascolo[2], Tim Coleman[5], Garry Barton[3,4], Lee Shepstone[3], Stephen Sutton[6], Toby Prevost[7], David Crane[8], Felix Greaves[9], Aimie Hope[1]

[1] Behavioural and Implementation Science Group, School of Health Sciences, University of East Anglia, UK

[2] Department of Computer Science and Technology, University of Cambridge, UK

[3] Norwich Clinical Trials Unit, University of East Anglia, UK

[4] Addiction Research Group, Norwich Medical School, University of East Anglia, UK

[5] Division of Primary Care, University of Nottingham, UK

[6] Behavioural Science Group, University of Cambridge, UK

[7] Nightingale-Saunders Clinical Trials & Epidemiology Unit, Kings College London, UK

[8] Department of Behavioural Science and Health, University College London, UK

[9] Department of Primary Care and Public Health, School of Public Health, Imperial College London, UK

* Corresponding author: Felix Naughton, Behavioural and Implementation Science Group, School of Health Sciences, University of East Anglia, Norwich, UK, NR4 7UL. Email: f.naughton@uea.ac.uk

Word count: 4,287

For peer review only - http://bmjopen.bmj.com/site/about/guidelines.xhtml

ABSTRACT

Introduction A lapse (any smoking) early in a smoking cessation attempt is strongly associated with reduced success. A substantial proportion of lapses are due to urges to smoke triggered by situational cues. Currently no available interventions proactively respond to such cues in real time. Quit Sense is a theory-guided Just-In-Time Adaptive Intervention (JITAI) smartphone app that uses a learning tool and smartphone sensing to provide in-the-moment tailored support to help smokers manage cue-induced urges to smoke. The primary aim of this randomised controlled trial (RCT) is to assess the feasibility of delivering a definitive online efficacy trial of Quit Sense.

Methods and analyses A two arm parallel-group RCT allocating smokers willing to make a quit attempt, recruited via online adverts, to usual care (referral to the NHS SmokeFree website) or usual care plus Quit Sense. Randomisation will be stratified by smoking rate (<16 vs. ≥ 16 cigarettes/day) and socioeconomic status (low vs. high). Recruitment, enrolment, baseline data collection, allocation and intervention delivery will be automated through the study website. Outcomes will be collected at 6 weeks and 6 months follow-up via the study website or telephone, and during app usage. The study aims to recruit 200 smokers to estimate key feasibility outcomes, the preliminary impact of Quit Sense, and potential cost-effectiveness, in addition to gaining insights on user views of the app through qualitative interviews.

Ethics and dissemination Ethics approval has been granted by the Wales NHS Research Ethics Committee 7 (19/WA/0361). The findings will be disseminated to the public, the funders, relevant practice and policy representatives and other researchers.

Trial registration: ISRCTN12326962.

Keywords: Smoking cessation, smartphone app, sensing, trial, online, just-in-time adaptive intervention

For peer review only - http://bmjopen.bmj.com/site/about/guidelines.xhtml

Article Summary

Strength and limitations of this study

- This is the first randomised controlled trial to evaluate a context-aware Just-In-Time Adaptive Intervention (JITAI) for smoking cessation.
- The study will identify key parameters to inform a definitive trial and will estimate intervention impact on biochemically validated abstinence from smoking.
- Recruitment, enrolment, baseline data collection, allocation, intervention delivery and most data collection during follow-up will be fully automated through the study website.
- There is potential for selection bias due to the requirement for participants to have internet access and an Android smartphone.

For peer review only

For peer review only - http://bmjopen.bmj.com/site/about/guidelines.xhtml

INTRODUCTION

Tobacco smoking is the second largest contributor of disease burden globally[1] and the largest in the UK.[2] While quitting reduces the risk of smoking related health problems and improves mental health,[3,4] the success rate remains low. Of the three million UK smokers attempting to quit annually, over 80% relapse within 12 months.[5]

Almost half of all lapses (any smoking) during a quit attempt are attributed to cravings triggered by environmental or situational cues[6] and lapses early in a quit attempt are highly predictive of longer-term relapse.[7–9] However, the most commonly used cessation medications do not alleviate cue-induced cravings.[10,11] Furthermore, while smokers are less likely to lapse if they use an effective cognitive and/or behavioural lapse prevention strategy[10,12,13] few do so.[12,14] There is a need for cessation interventions that effectively support the management of cue-induced cravings.

Unlike traditional cessation interventions smartphone apps have the advantage of portability and could therefore better assist with cue-induced cravings as they occur. Some mobile phone-based interventions have features specifically aimed at reducing lapse risk. Such support can be triggered by random Ecological Momentary Assessment (EMA) prompts[15] (where the smartphone prompts the user to complete assessment questions); or it can take the form of on-demand (i.e., user-initiated) requests for craving support.[16,17] However, given the rapid time to lapse after craving onset[10] and that many on-demand craving tools are seldom used beyond a first try,[17,18] these kinds of user-initiated features may still miss many craving episodes.

To address such limitations in current digital support we developed Quit Sense, a digital intervention which predicts and adapts to smoking risk in real time[19] (a Just-In-Time Adaptive Intervention). Quit Sense is a theory-guided smoking cessation tool that delivers 'context aware' lapse prevention support primarily using smartphone location sensing. It has demonstrated feasibility in delivering 'in the moment' support and acceptability among users.[20] We are now conducting a feasibility randomised controlled trial of Quit Sense to inform the design parameters of a future randomised control trial (RCT) and to provide estimates of intervention impact.

For peer review only - http://bmjopen.bmj.com/site/about/guidelines.xhtml

This trial is being conducted during the COVID-19 pandemic and as such different contextual variables may impact on study participants during their quit attempts (e.g., self-isolation, remote working) compared to before the COVID-19 pandemic. Through qualitative interviews and app recorded feedback this feasibility trial will capture participant views of the acceptability of the app and study, including how these are impacted by any COVID-19 restrictions.

Objectives

In this feasibility trial we aim to estimate:

a) Completion rates for the anticipated primary outcome for a full trial
b) Usual care arm cessation rate
c) Cost of recruitment using online advertising
d) Rates of app installation and use, and support delivery fidelity
e) Completion of smoking cessation-related resource use and quality of life data
f) Participant views of the app, as part of a qualitative process evaluation
g) Intervention effect on anticipated primary outcome
h) Intervention effect on hypothesised mechanisms of action of app at 6 weeks post-enrolment

METHODS AND ANALYSIS

Study design

A two-arm parallel-group randomised controlled feasibility trial, allocating smokers to either ‘usual care’ (link to NHS SmokeFree website)[21] or the intervention arm who receive ‘usual care’ plus access to the Quit Sense app.

Additionally, there is a nested qualitative process evaluation with a subsample of control and intervention participants to 1) assess intervention experiences and how these might help explain causal pathway(s) towards smoking behaviour change (intervention arm only)[22] and 2) asses experiences of study participation.

Additional information on study configuration can be found in Appendix 1.

For peer review only - http://bmjopen.bmj.com/site/about/guidelines.xhtml

For peer review only

Table 1 Schedule of participant assessments and intervention delivery

	STUDY PERIOD			
	Enrolment (online)	Allocation (online)	Post-allocation (online or telephone)	Close-out (online or telephone)
TIMEPOINT	0	0	*6 weeks FU (from enrolment)	**6 months FU (from enrolment)
ENROLMENT:				
Eligibility screen	X			
Informed consent	X			
Allocation		X		
INTERVENTIONS:				
Usual care (link to www.smokefree.nhs.uk)		X		
Quit Sense app + usual care		X		
ASSESSMENTS:				
Demographics	X			
Use of smartphone and apps	X			X
Smoking behaviour and dependence	X			X
Smoking beliefs	X			X
Cessation self-efficacy	X		X	X
Strength and frequency of urges to smoke	X		X	X

For peer review only - http://bmjopen.bmj.com/site/about/guidelines.xhtml

Automaticity and associative processes subscales of the WISDM-37	X		X	
EQ-5D-5L	X			X
Smoking lapse incidence			X	
Smoking abstinence outcomes			X	X
Use of lapse prevention strategies			X	
Smoking cessation resource use				X
Views on the app (Quit Sense arm only)				X
Assessment of Tobacco Exposure (postal saliva sample)				X

*Follow-up at 6 weeks (one month, plus 2 weeks to cover the likely quitting period/phase 1 of the app).

** Follow-up will be scheduled approximately 6 months post enrolment in the study, allowing for an additional 2 weeks to cover the likely quitting period (phase 1 of the app). Although in practice this will be 6 months plus 2 weeks, we will refer to it simply as the '6-month follow-up' for convenience.

Participants

Eligibility criteria

Participants will be smokers aged 16 years plus; currently smoke at least 7 cigarettes per week; willing to make a quit attempt within 14 days of enrolling; have primary use of an Android smartphone (version 5.0 and above) and will be resident in England. Participants must not have previously participated in this trial.

For peer review only - http://bmjopen.bmj.com/site/about/guidelines.xhtml

Study setting

Participants will complete screening, consent, baseline and follow-up measures online. This online design matches the ‘real world’ setting of smartphone app uptake.

Online recruitment

Recruitment is through paid-for online advertisements with Google Search and social media (Facebook and Instagram), limited to England-based IP addresses and targeted at Android devices. Advertisements are managed by ‘Nativve’,[23] specialists in digital marketing for research study recruitment. To maximise participation rate efficiency and in accordance with PPI recommendations, different adverts will be tested using A/B experiments while recruitment is live. People clicking on adverts will be directed to the study website and provided with study information including a downloadable ‘Participant Information Sheet.’ The screening and consent procedures are online with an e-signature confirming consent (Table 1, Appendix 2). After completing an online baseline questionnaire, participants will be randomly allocated to usual care or usual care plus Quit Sense. As described in Figure 1 and informed by a prior text message smoking cessation intervention,[24] we estimate that ~6,625 unique visitors are required to achieve a sample size of 200.

The distribution of socioeconomic status (SES) during recruitment will be monitored. Low SES is defined as individuals with a semi-routine or routine and manual occupation, class 5 in the National Statistics Socio-Economic Classification (NS-SEC),[25] or who have never worked or are long-term unemployed. Targeted advertising will be used to increase low SES representation if less than 45% of our sample are categorised as low SES when 35% of the target sample is reached.

Procedure

Both arms will automatically receive a link to NHS SmokeFree via SMS after enrolment. Only the Quit Sense arm will receive an SMS with an app link and activation code. Use of the activation code will be monitored. Participants who do not initiate the app will be sent an SMS reminder 3 days post enrolment and then five days post enrolment asking to identify a reason for not initiating the app from a predefined list.

For peer review only - http://bmjopen.bmj.com/site/about/guidelines.xhtml

To attempt to increase response rates all participants will receive an SMS prior to receiving links to follow-up questionnaires. Those who do not respond to the SMS with a survey link will be telephoned. Once the six-week questionnaire is completed, a subsample of participants from both arms (approximately 3:1 ratio between Quit Sense and usual care) will be invited to a telephone interview to gather data on their experiences of the app (intervention only) and of participating in the study. Where practicable we will use purposive sampling to interview a mixture of high and low SES abstainers and smokers, and for Quit Sense participants based on varying rates of app engagement. We anticipate interviewing 20 participants, depending on when data saturation is reached.

Six months and two weeks after enrolling (allowing for a two-week quitting preparation period for Quit Sense participants; henceforth referred to as the 'six-month' follow-up), all participants will be asked via SMS to complete the final questionnaire. Participants reporting abstinence in the previous 7 days at follow up will be posted a saliva sample kit to be returned to ABS laboratories, UK.

Participants will receive vouchers on completion of the 6-week (£5) and 6-month (£10) surveys, return of the saliva sample (£5) and for participating in an interview (£20).
As suggested by our PPI panel, all participants will receive a postcard and SMS to thank them and emphasise their importance to the study.

Criteria for discontinuing or modifying allocated interventions

Unless a participant withdraws from the study, they will be followed-up for data collection regardless of their level of engagement or smoking status. Participants who discontinue using the app for any reason other than withdrawal, will remain in the trial for the purpose of follow-up and data analysis.

For peer review only - http://bmjopen.bmj.com/site/about/guidelines.xhtml

Interventions

Usual Care Arm

Usual care is a web-link (NHS SmokeFree) sent by SMS. We consider this equivalent to 'usual care' for an online population. SmokeFree provides access and signposting to digital, telephone and in-person cessation support in England. At the time of writing the support offered includes information and links to six main types of support; support from a cessation advisor (via a Stop Smoking Service), the SmokeFree National telephone helpline, an online chat facility with an advisor, an email cessation programme, an SMS text message programme and the NHS SmokeFree smartphone app (28-day programme, non-tailored and not including proactive support delivery for managing urges to smoke).

Quit Sense app Arm

Quit Sense is a context-aware smartphone app designed for smokers wanting to quit. It is informed by learning theory (LT)[26,27] and two theory-guided SMS text message systems,[17,28] which are in turn informed by Social Cognitive Theory (SCT).[29] Quit Sense targets three key determinants that map onto LT and six mapping onto SCT: learned associations between smoking and an individual's physical environment (LT); learned associations between smoking and mood triggered by the environment (LT); the presence of other smokers (LT, SCT); awareness of smoking triggers (antecedents) (SCT); outcome expectancies of a lapse (SCT); goal/intention for complete abstinence (SCT); self-efficacy in resisting urges to smoke (SCT); knowledge of and self-efficacy for lapse prevention strategy use (SCT). Twenty-one corresponding Behaviour Change Techniques (BCTs)[30] are used to target these determinants.

The main feature is 'Geofence-Triggered Support' (GTS), which is orientated around three app stages:

- Stage 1 (training): The user sets a quit date (default suggestion 7 days) and trains the app to learn about their smoking behaviour. This requires the user to report in the app each smoking episode and the situational context in real time (stress, mood, urge strength, setting description [home, working, home-working, socialising, other], presence of other smokers), while the app records geolocation. If a user reports smoking more than once in the same location (as defined by the app) a geofence (a

For peer review only - http://bmjopen.bmj.com/site/about/guidelines.xhtml

circular virtual perimeter) is created representing a high-risk area. Location monitoring precision varies depending on the availability of GPS/WiFi/network (range ~ 5-50 m).

- Stage 2 (28-day abstinence challenge): Post quit date, the app monitors the user's location. A support message is triggered a minimum of 1 minute after the user enters a geofence zone (typical range 1-15 mins, depending on operating system). Messages are individually tailored using Stage 1 context information and using any stage 2 smoking reports, providing support to help users avoid or cope with location specific triggers. Further messages are triggered after each 3-hour interval of remaining in that location (default between 8am-9.30pm or as defined by the user). This also applies to 'home-working' messages. However, if the user has not reported 'home-working' within the time window (2-3 hours) in which the message is to be delivered, a 'home' message will be delivered instead. This is because 'time' of reporting is an additional requirement (to 'location') for home-working messages to be triggered.
- Stage 3 (maintaining abstinence): The app continues to deliver GTS for 2 further months but reduces the frequency by one-half every month. After 3 months post-quit date, the GTS support stops, unless the user opts to switch it back on or restart their quit attempt.

Quit Sense has six additional features (relevant to all stages, unless specified otherwise):

1. Feedback is provided after each smoking report. In Stage 1 this is tailored to nine characteristics collected during app initiation. Messages focus on enhancing quitting preparation, motivation and self-efficacy. In Stage 2, feedback is non-tailored and orientated around lapse and relapse prevention.
2. An 'End of Day' (EoD) survey that users are prompted to complete, recording cigarettes smoked that day, cravings[31] and self-efficacy[28,29] (Stages 1 and 2 only). Feedback is provided after each EoD survey, including encouragement to log smoking behaviour if there is a discrepancy between the survey and real time reports.
3. A 'my profile' screen including number of days quit, money saved (based on smoking rate and reported cost), a calendar showing a heat map and 'smileys' (use of yellow, grey and blue to denote values with corresponding happy, neutral and sad smileys) for smoking, cravings and self-efficacy for each EoD survey

For peer review only - http://bmjopen.bmj.com/site/about/guidelines.xhtml

completed. In addition, smoking pattern feedback is provided using graphical and written summaries for smoking triggers based on the user's historical reporting.

4. A 'library' of quitting advice split into six categories: 'getting ready', 'boost your motivation', effects of smoking and quitting', 'early days of quitting', 'staying quit', 'smoking after your quit date'. Users can write and submit their own support messages.
5. Scheduled non-tailored daily support messages delivered each morning orientated around the quit date (Stages 1 and 2 only) – targeting outcome expectancies, motivation, preparation, self-efficacy (three sets of different messages to prevent message duplication in successive attempts).
6. The option for the user to reset their quit date in Stage 1, if they are not feeling ready, or Stage 2 if they relapse. Relapse is defined as multiple smoking episodes that are reported over two days during a quit attempt (either through smoking episode logs or the EoD survey). If this occurs, users are invited to either re-commit and continue or reset their quit date and return to Stage 1.

Qualitative process evaluation

The aims of the qualitative process evaluation are:

1. To gather user experiences of app usage in order to inform app optimisation.
2. To understand participant experiences of participation and the unique contexts in which participants are attempting to quit.

Interview questions will probe for in-depth descriptions of intervention participants' experiences of app usage including which types of support they liked most and least, the timing of support in relation to experienced need for support, and views on the features used. Control participants will be asked about their experience of trial participation. Both groups will be asked about their use of or interest in other smoking cessation aids and support and contextual details such as mandated movement restrictions and family setting. In addition, the Quit Sense app includes an in-built audio record feature so that users can leave feedback. The study will therefore also assess the feasibility of a user-initiated process evaluation approach where data is collected with high ecologically validity. Any relevant data submitted, will be analysed alongside interview data, potentially providing a broader range of views from a more user-controlled perspective. Transcriptions from interviews or app recordings will be

For peer review only - http://bmjopen.bmj.com/site/about/guidelines.xhtml

anonymised. Recordings will be deleted after transcription, or if not fully transcribed, after audio coding for analysis.

Database

Study data are collected and managed using REDcap (Research Electronic Data Capture) tools hosted at Norwich Clinical Trials Unit (NCTU). REDcap is a secure, web-based software platform supporting data capture for research studies.[32,33] Further details on data management can be found in the online Supplementary File.

Outcomes

Feasibility Outcomes

As this is a feasibility trial there is no primary outcome. Feasibility outcomes will be used to estimate key parameters to inform a future trial,[34] and include:

i. Completeness of the anticipated primary outcome for a future definitive trial (6-month self-reported abstinence with biochemical validation; see smoking outcomes section).
ii. Abstinence rate of usual care arm, using the anticipated primary outcome for a future definitive trial.
iii. Cost per recruit, based on advertising costs.
iv. Rates of app installation use and support delivery fidelity.
v. Completion of smoking cessation-related resource use, including usual care use, and quality of life (EQ-5D-5L)[35] data.
vi. Hypothesised mechanisms of action of Quit Sense.
vii. Participant experiences and feedback on app usage.

Smoking and related outcomes

Smoking and related outcomes will provide preliminary information about the intervention's impact. The main abstinence outcome for which we will monitor completeness of ascertainment is based on the Russell standard.[36] Abstinence will be defined as: self-reported abstinence in the previous 6 months allowing for no more than five cigarettes and not smoking in the previous week, biochemically validated by a saliva cotinine concentration of less than 10 ng/ml[36,37] and for those using nicotine substitution (e.g. Nicotine

For peer review only - http://bmjopen.bmj.com/site/about/guidelines.xhtml

Replacement Therapy, or e-cigarettes) an anabasine concentration of less than 0.2 ng/ml , based on feedback from our testing laboratory.[37,38] Thresholds will be reviewed prior to analysis in case of changes in guidance or relevant evidence. Given recent evidence that some e-liquid, believed to be that made outside of the UK, can contain anabasine,[37] we will monitor the proportion of e-cigarette users who report abstinence from smoking but have an anabasine level above the chosen threshold.

Smoking cessation experts highlight the value of additional smoking outcomes based on different time periods of abstinence and time-points.[39] We will therefore measure 7-day point prevalence abstinence at 6 months follow-up (self-report and biochemically verified) and 7-day point prevalence abstinence at 6 weeks follow-up (self-report).

We will collect the following hypothesised mechanisms of action at 6-week follow-up:

- Lapse incidence (any smoking, even a puff) since the initial quit attempt (if made),[6] which is highly predictive of relapse.[7–9]
- Lapse prevention strategy use,[14] which is associated with lapse prevention.[10]
- Self-efficacy,[28,29] which prospectively predicts lapse and relapse to smoking.[40]
- The Strength of Urges to Smoke (SUTS) measure,[31] which prospectively predicts abstinence and is superior to other urge-measures in doing so,[41] and Frequency of Urges to Smoke (FUTS).[41]
- Automaticity and associative processes subscales from the Wisconsin Inventory of Smoking Dependence Motives (WISDM-37).[42]

Sample size

In line with recommendations that feasibility studies with binary outcome measures recruit at least 60 participants per group (minimum of 120) and a maximum of 100 per group, the proposed sample size for this trial is 200 smokers (100 per arm).[43] Participants will be randomised to usual care or Quit Sense arms on a 1:1 ratio. This sample size will enable key parameters to inform a future definitive trial to be estimable within the following precision (defined as the 95% confidence interval [CI] half-width):

- Primary outcome completion: we estimate a follow-up rate for self-reported smoking status at 6 months of 80%, with precision of +/- 6%.[44–46] For

For peer review only - http://bmjopen.bmj.com/site/about/guidelines.xhtml

biochemical validation, we estimate that 75% of participants reporting abstinence will return a saliva sample by post, with precision of +/- 22%.[28,45]

- Cessation rate in usual care arm: we estimate an abstinence rate of 5% at 6 months follow-up, providing precision of +/- 4%.[47]
- App installation and initiation: we estimate that 85% of intervention participants will install the app, with precision of +/- 7%.[48]
- App engagement – in our acceptability study, 71% used the app for more than one week (a timeframe deemed meaningful by our PPI panel). With a similar rate in the trial, precision would be +/- 9%. This is in line with other studies.[48]

For peer review only

Randomisation

Sequence generation, allocation and blinding

Randomisation will be stratified by smoking rate (<16 vs. ≥ 16 cigarettes/day; based on mean smoking rates from trials recruiting smokers online)[45,49] and socioeconomic status (low vs. high), based on the National Statistics Socio Economic Classification (NS-SEC) self-coded method.[50] Allocation sequences will be generated by a computer-based random number generator using random permuted blocks (varying block sizes) (using REDcap). Randomisation will be integrated into the enrolment process on the study website. The system will not permit a participant to enrol twice with the same mobile number or email address. As allocation will be integrated into the study website, the allocation sequence will be concealed from participants until assignment and concealed from members of the trial team, except the statistician, developers of the study database and the research associate supporting trial delivery.

Data analysis

The feasibility and acceptability of all measures will be assessed by their level of completeness and via interviews and app audio data.

For peer review only - http://bmjopen.bmj.com/site/about/guidelines.xhtml

Outcomes

Completeness of the anticipated primary outcome for a future trial (outcome i.) and the abstinence rate using this anticipated primary outcome for the usual care arm (outcome ii.) will be described as proportions with 95% CIs, and translated into interpretable probabilities using the Bayesian approach relevant in preliminary trials with the objective of powering the definitive trial.

Cost per recruit (outcome iii.), rates of app installation (outcome iv.) and completion of smoking cessation-related resource use and quality of life (EQ-5D-5L) (outcome v.) will be described using summary statistics with 95% CIs.

We will estimate the preliminary intervention effect on abstinence, lapse incidence and use of lapse prevention strategies, using multiple logistic regression, providing odds ratios with 95% CIs, while adjusting for any potential baseline confounders defined by the Statistical Analysis Plan (SAP). For the estimated intervention effect on abstinence we will assume missing=smoking.[36] To estimate intervention effects on self-efficacy, urges and smoking dependence motives, we will use ANCOVA models involving adjustment for the baseline score and for pre-defined potential confounders, primarily using complete cases only. Missing outcome assumptions will be assessed through sensitivity analyses which will be defined in the SAP.

Economic evaluation

We aim to collect data (baseline and 6-months follow-up) that will enable us to inform the decision as to how cost (based on resource use data) and benefit (in terms of quality of life [QoL]) data are estimated in any future more definitive study. Completion rates will thereby be estimated.

In terms of costs, we will estimate those associated with the intervention (e.g. app maintenance) and smoking related costs to both the individual (e.g. nicotine replacement therapy) and the NHS (e.g. NHS stop smoking services/GP visits). In line with NICE guidance,[51] QoL will be measured via the EQ-5D-5L[35] as this enables the calculation of

For peer review only - http://bmjopen.bmj.com/site/about/guidelines.xhtml

QALY (Quality Adjusted Life Year) scores. Appropriate unit costs (e.g.[52]) will also be attached to all items of resource-use in order to identify major cost drivers.

Based on the intention to treat approach, a preliminary cost-effectiveness analysis will be performed. In the base-case analysis, costs will be estimated from the viewpoint of the NHS and personal social services.[51] We will estimate the mean incremental cost and mean QALY gain associated with Quit Sense compared to usual care. Assuming dominance does not occur (where one intervention is both more costly and less effective), then the incremental cost effectiveness ratio (incremental cost/QALY gain) will be estimated and assessed in relation to a range of cost-effectiveness thresholds (e.g. £20,000 to £30,000 per QALY),[51] in order to provide a preliminary assessment of whether Quit Sense constitutes value for money. The associated level of uncertainty will also be estimated.

Qualitative process evaluation data collection and analysis

Interviews will be audio-recorded and transcribed intelligent verbatim. App audio-recordings may be transcribed depending on quality and resources. If transcription is unfeasible, coding of audio files will be completed.

We will undertake an inductive thematic analysis of the first 3-4 interview transcripts,[53] develop a coding framework from this, and then code remaining data. We will check coding consistency through the independent coding of 10% of the data, with a further 10% if consistency is unsatisfactory. We will continue refining until the themes are agreed. Analysis of process evaluation data will focus on identifying key themes associated with using the app and views and experiences of using other relevant apps. A second stage of analysis will work with descriptions of experiences of lapse or lapse avoidance in the context of the app and considering unique participant contexts. These will be reported as vignettes and used to explore participant experiences and to identify mechanisms of action and casual pathways towards behaviour change.

Patient and Public Involvement

We have a patient and public involvement (PPI) panel of 4 former smokers who have contributed to study design including the recruitment strategy, wording of SMS messages,

For peer review only - http://bmjopen.bmj.com/site/about/guidelines.xhtml

study information, the qualitative evaluation, the design of the study logo, and the function and features of the Quit Sense app and support it delivers. The PPI panel is represented on the Trial Steering Committee.

DISCUSSION

A feasibility trial of Quit Sense will identify the conditions necessary for a definitive trial, including for successful study design and data collection. This will largely be determined by whether: the recruitment strategy is able to produce the required sample size and participant retention rates; the rate of app installation; completion rates for follow-up and the anticipated primary outcome for a full trial; and participant views of the acceptability of the app and study. A potential future RCT will enable us to establish the extent to which the Quit Sense app is effective and cost effective relative to the usual provision of online stop smoking support.

This remotely delivered and largely automated trial is being conducted during the COVID-19 pandemic, a time when health protection measures are restricting or preventing many everyday behaviours. As such, there are likely to be different contextual variables impacting on study participants (e.g., self-isolation, remote working, limited opportunities to socialise, etc.) compared to before the pandemic. The Quit Sense app can learn about such situational factors because of the initial training phase where the user reports on their smoking behaviour. Furthermore, we hope to capture in-depth insights into the unique contexts in which participants are attempting to make their quit attempts through telephone interviews and the app's inbuilt audio-record feature.

The potential of intelligent and 'always on' digital cessation tools in providing on-hand effective and low cost support means that they are likely to be of growing importance in enhancing chances of successful quitting.[54] However, measures are needed to realise the potential of next generation digital cessation interventions most of which remain untested.[15] Where app evaluations have been undertaken results show that evidence-based smoking cessation strategies are underutilized,[55] that many use a limited number of behaviour change techniques,[56,57] and that few utilise features such as geolocation or just-in-time sensors.[56] It is crucial, therefore, that their effectiveness and cost effectiveness are established, particularly through use of randomised control trials.[58]

For peer review only - http://bmjopen.bmj.com/site/about/guidelines.xhtml

Quit Sense has been developed to address many of the current limitations in digital support. The app is distinct from other cessation support available due to its ability to identify location-based high-risk locations using smartphone sensing to provide timely theory-guided and tailored 'in-the-moment' support. Quit Sense has been developed to help smokers avoid or manage urges to smoke triggered by cues within these high-risk locations. This is done through the provision of lapse prevention strategies, outcome expectancy management and motivational and self-efficacy enhancement. Ultimately this support aims to help extinguish learned associations between smoking and a person's physical, social and psychological environment in order to reduce future relapse risk.

For peer review only

For peer review only - http://bmjopen.bmj.com/site/about/guidelines.xhtml

Author affiliations

University of East Anglia
Norwich Clinical Trials Unit
University of Nottingham

Author Contributions

FN, CN, CM, TC, GB, SS, TP, DC and FG conceived the study project and obtained project funding. FN led on app design, CB led on app development and AH and CB contributed to app design. FN and AH led on protocol development and drafted the manuscript.
CN contributed to the qualitative process evaluation section. GB led on the health economics section. LS contributed to the statistical analysis section. JH contributed to regulatory and Clinical Trials Unit related content. All authors contributed to the preparation and drafting of the manuscript.

Funding

This study is funded by the National Institute for Health Research (NIHR) Public Health Research Programme (17/92/31). Tim Coleman is an NIHR Senior Investigator.

Disclaimer

The views expressed are those of the authors and not necessarily those of the NIHR or the Department of Health and Social Care.

Competing interests

FG is also employed by NICE. DC is founder and CEO of a smoking cessation app called Smoke Free (23 Limited), which has no connection to Quit Sense.

Patient consent for publication

Not required.

Ethics approval

Ethical approval for this study was granted on the 28th of January 2020 (Wales REC 7, reference 19/WA/0361, IRAS project ID 270432).

Providence and peer review

Not commissioned, peer reviewed for ethical and funding approval prior to submission.

For peer review only - http://bmjopen.bmj.com/site/about/guidelines.xhtml

For peer review only

Open access

There is no data associated with this manuscript.

Protocol version 1.3 Date 15/12/2020

Trial sponsor

Graham Horne,

Research and Innovation Services,

University of East Anglia,

Norwich Research Park,

NR4 7TJ

research.sponsor@uea.ac.uk

01603 591477

Trial status

Recruiting

For peer review only - http://bmjopen.bmj.com/site/about/guidelines.xhtml

References

1 Roser M, Ritchie H. Burden of Disease. 2016. https://ourworldindata.org/burden-of-disease (accessed 27 Apr 2020).

2 Murray CJ, Richards MA, Newton JN, Fenton KA, Anderson HR, Atkinson C, Bennett D, Bernabé E, Blencowe H, Bourne R, Braithwaite T, Brayne C, Bruce NG, Brugha TS, Burney P, Dherani M, Dolk H, Edmond K, Ezzati M, Flaxman AD, Fleming TD, Freedman G, Gunnell D DA. UK health performance: findings of the Global Burden of Disease Study 2010. *Lancet* 2013;**381**:997–1020. doi:10.1016/S0140-6736(13)60355-4

3 National Center for Chronic Disease Prevention and Health Promotion (US) Office on Smoking and Health. The Health Consequences of Smoking - 50 years of Progress: A report of the Surgeon General. 2014. https://www.ncbi.nlm.nih.gov/books/NBK179276/

4 Taylor G, McNeill A, Girling A, Farley A, Lindson-Hawley N AP. Change in mental health after smoking cessation: Systematic review and meta-analysis. *BMJ* 2014;**348**:2216. doi:10.1136/bmj.g2216

5 West, R, Brown J. Smoking Toolkit Study. 2017. http://www.smokinginengland.info/ (accessed 27 Apr 2020).

6 Shiffman S, Paty JA, Gnys M, Kassel JA, Hickcox M. First lapses to smoking: within-subjects analysis of real-time reports. *J Consult Clin Psychol* 1996;**64**:366-379. doi:10.1037//0022-006x.64.2.366

7 Kenford SL, Fiore MC, Jorenby DE, Smith SS, Wetter D, Baker TB. Predicting Smoking Cessation. Who Will Quit With and Without the Nicotine Patch. *JAMA* 1994;**8**:589-94.https://www.ncbi.nlm.nih.gov/pubmed/8301790

8 Deiches DF, Baker TB, Lanza S, Piper ME. Early Lapses in a Cessation Attempt: Lapse Contexts, Cessation Success, and Predictors of Early Lapse. *Nicotine Tob Res* 2013;**15**:1883–91. doi:10.1093/ntr/ntt074

9 Shadel WG, Martino SC, Setodji C, Cervone D, Witkiewitz K, Beckjord EB, Scharf D, Shih R. Lapse-induced surges in craving influence relapse in adult smokers: an experimental investigation. *Health Psychol* 2011;**30**:588–96.

10 Ferguson SG, Shiffman S. The relevance and treatment of cue-induced cravings in tobacco dependence. *J Subst Abuse Treat* 2009;**36**:235–43. doi:10.1016/j.jsat.2008.06.005

For peer review only - http://bmjopen.bmj.com/site/about/guidelines.xhtml

11 Ferguson SG, Shiffman S. Effect of high-dose nicotine patch on craving and negative affect leading up to lapse episodes. *Psychopharmacology (Berl)* 2014;**231**:2595–602. doi:10.1007/s00213-013-3429-6

12 Brodbeck J, Bachmann MS, Znoj H. Distinct Coping Strategies Differentially Predict Urge Levels and Lapses in a Smoking Cessation Attempt. *Addict Behav* 2013;**38**:2224–9. doi:10.1016/j.addbeh.2013.02.001

13 O'Connell KA, Hosein VL, Schwartz JE. Thinking and/or doing as strategies for resisting smoking. *Res Nurs Heal* 2006;**29**:533–42. doi:10.1002/nur.20151

14 Naughton F, McEwen A, Sutton S. Use and effectiveness of lapse prevention strategies among pregnant smokers. *J Health Psychol* 2015;**20**:1427–33. doi:10.1177/1359105313512878

15 Hébert ET, Ra CK, Alexander AC, *et al.* A Mobile Just-in-Time Adaptive Intervention for Smoking Cessation: Pilot Randomized Controlled Trial. *J Med Internet Res* 2020;**22**:e16907. doi:10.2196/16907

16 Free C, Knight R, Robertson S, *et al.* Smoking cessation support delivered via mobile phone text messaging (txt2stop): A single-blind, randomised trial. *Lancet* 2011;**378**:49–55. doi:10.1016/S0140-6736(11)60701-0

17 Naughton F, Jamison J, Boase S, *et al.* Randomized controlled trial to assess the short-term effectiveness of tailored web- and text-based facilitation of smoking cessation in primary care (iQuit in Practice). *Addiction* 2014;**109**:1184–93. doi:10.1111/add.12556

18 Devries KM, Kenward MG, Free CJ. Preventing Smoking Relapse Using Text Messages: Analysis of Data From the txt2stop Trial. *Nicotine Tob Res* 2012;**15**:77–82. doi:https://doi.org/10.1093/ntr/nts086

19 Naughton F. Delivering 'Just-in-Time' smoking cessation support via mobile phones: Current knowledge and future directions. *Nicotine Tob Res* 2016;**19**:379–83 doi: 10.1093/ntr/ntw143

20 Naughton F, Hopewell S, Lathia N, *et al.* A Context-Sensing Mobile Phone App (Q Sense) for Smoking Cessation: A Mixed-Methods Study. *JMIR mHealth uHealth* 2016;**4**:e106. doi:10.2196/mhealth.5787

21 NHS Digital. SmokeFree. Online. 2020 https://www.nhs.uk/smokefree

22 Moore GF, Audrey S, Barker M, *et al.* Process evaluation of complex interventions: Medical Research Council guidance. *BMJ* 2015;**350**:1–7. doi:10.1136/bmj.h1258

23 Nativve: Digital Marketing Specialists. 2020. https://www.nativve.com/

24 Emery JL, Coleman T, Sutton S, Cooper S, Leonardi-Bee J, Jones M, Naughton F.

For peer review only - http://bmjopen.bmj.com/site/about/guidelines.xhtml

Uptake of Tailored Text Message Smoking Cessation Support in Pregnancy When Advertised on the Internet (MiQuit): Observational Study. *J Med Internet Res* 2018;**20**:e146. doi:10.2196/jmir.8525.

25 Office for National Statistics. The National Statistics Socio-economic classification (NS-SEC). Online. 2016. https://www.ons.gov.uk/methodology/classificationsandstandards/otherclassifications/thenationalstatisticssocioeconomicclassificationnssecrebasedonsoc2010

26 Pavlov I. *Conditioned reflexes*. London and New York: Oxford University Press 1927.

27 Skinner B. *The behavior of organisams: An experimental analysis*. New York: Appleton-Century 1938.

28 Naughton F, Prevost AT, Gilbert H, Sutton S. Randomised controlled trial evaluation of a tailored leaflet and SMS text message self-help intervention for pregnant smokers. *Nicotine Tob Res* 2012;**14**:569–77. doi::10.1093/ntr/ntr254

29 Bandura A. *Social foundations of thought and action: A social cognitive theory*. Englewood Cliffs: Prentice-Hall 1986.

30 Michie S, Richardson M, Johnston M, Abraham C, Francis J, Hardeman W, Eccles MP, Cane J, Wood CE. The behavior change technique taxonomy (v1) of 93 hierarchically clustered techniques: building an international consensus for the reporting of behavior change interventions. *Ann Behav Med* 2013;**46**:81–95. doi:10.1007/s12160-013-9486-6

31 West R, Hajek P. Evaluation of the Mood and Physical Symptoms Scale (MPSS) to Assess Cigarette Withdrawal. *Psychopharmacol* 2004;**177**:195–9. doi:10.1007/s00213-004-1923-6

32 Harris PA, Taylor R, Thielke R, Payne J, Gonzalez N, Conde JG. Research electronic data capture (REDCap)--a metadata-driven methodology and workflow process for providing translational research informatics support. *J Biomed Inf* 2009;**42**:377–81. doi:10.1016/j.jbi.2008.08.010

33 Harris PA, Taylor R, Minor BL, Elliott V, Fernandez M, O'Neal L, McLeod L, Delacqua G, Delacqua F, Kirby J, Duda SN, REDCap Consortium. The REDCap consortium: Building an international community of software platform partners. *J Biomed Inform* 2019;**95**. doi:10.1016/j.jbi.2019.103208.

34 Craig P, Dieppe P, Macintyre S, *et al.* Developing and evaluating complex interventions: The new Medical Research Council guidance. *BMJ* 2008;**337**:979–83. doi:10.1136/bmj.a1655

For peer review only - http://bmjopen.bmj.com/site/about/guidelines.xhtml

35 Herdman M, Gudex C, Lloyd A, *et al.* Development and preliminary testing of the new five-level version of EQ-5D (EQ-5D-5L). *Qual Life Res* 2011;**20**:1727–36. doi:10.1007/s11136-011-9903-x

36 West R, Hajek P, Stead L, Stapleton J. Outcome Criteria in Smoking Cessation Trials: Proposal for a Common Standard. *Addiction* 2005;**100**:299–303. doi:10.1111/j.1360-0443.2004.00995.x.

37 Benowitz NL, Bernert JT, Foulds J, Hecht SS, Jacob P, Jarvis MJ, Joseph A, Oncken C, Piper ME. Biochemical Verification of Tobacco Use and Abstinence: 2019 Update. *Nicotine Tob Res* doi:10.1093/ntr/ntz132

38 Jacob P, Hatsukami D, Severson H, Hall S, Yu L, Benowitz N. Anabasine and anatabine as biomarkers for tobacco use during nicotine replacement therapy. *Cancer Epidemiol Biomarkers Prev* 2002;**11**:1668–73.

39 Cheung KL, De Ruijter D, Hiligsmann M, *et al.* Exploring consensus on how to measure smoking cessation. A Delphi study. *BMC Public Health* 2017;**17**:1–10. doi:10.1186/s12889-017-4902-7

40 Gwaltney CJ, Shiffman S, Balabanis MH, Paty JA. Dynamic self-efficacy and outcome expectancies: prediction of smoking lapse and relapse. *J Abnorm Psychol* 2005;**114**:661–75. doi:10.1037/0021-843X.114.4.661

41 Fidler JA, Shahab LS, West R. Strength of urges to smoke as a measure of severity of cigarette dependence: Comparison with the Fagerstrom Test for Nicotine Dependence and its components. *Addiction* 2010;**106**:631–8. doi:10.1111/j.1360-0443.2010.03226.x

42 Smith SS, Piper ME, Bolt DM, Fiore MC, Wetter DW, Cinciripini PM, Baker TB. Development of the Brief Wisconsin Inventory of Smoking Dependence Motives. *Nicotine Tob Res* 2010;**12**:489–99. doi:10.1093/ntr/ntq032

43 Teare MD, Dimairo M, Shephard N, *et al.* Sample size requirements to estimate key design parameters from external pilot randomised controlled trials: a simulation study. *Trials* 2014;**15**:264.

44 Bricker JB, Mull KE, Kientz JA. Randomized, controlled pilot trial of a smartphone app for smoking cessation using acceptance and commitment therapy. *Drug Alcohol Depend* 2014;**143**:87–94. doi:doi:10.1016/j.drugalcdep.2014.07.006.

45 Brown J, Michie S, Geraghty AWA, Yardley L, Gardner B, Shahab L, Stapleton JA, West R. Internet-based intervention for smoking cessation (StopAdvisor) in people with low and high socioeconomic status: a randomised controlled trial. *Lancet Respir*

For peer review only - http://bmjopen.bmj.com/site/about/guidelines.xhtml

Med 2014;**2**:997–1006. doi:10.1016/S2213-2600(14)70195-X

46 Taylor GMJ, Dalili MN, Semwal M, Civljak M, Sheikh A, Car J. Internet-based interventions for smoking cessation. Cochrane Database Syst Rev. *Cochrane Database Syst Rev* 2017;9(9) doi:10.1002/14651858.CD007078.pub5

47 Whittaker R, Mcrobbie H, Bullen C, Rodgers A, Gu Y. Mobile phone-based interventions for smoking cessation. *Cochrane Database Syst Rev* 2016; 10;4(4). doi:10.1002/14651858.CD006611.pub4

48 Iacoviello BM, Steinerman JR, Klein DB, *et al.* Clickotine, A Personalized Smartphone App for Smoking Cessation: Initial Evaluation. *JMIR mHealth uHealth* 2017;**5**:e56. doi:10.2196/mhealth.7226

49 Mason D, Gilbert H, Sutton S. Effectiveness of web-based tailored smoking cessation advice reports (iQuit): A randomized trial. *Addiction* 2012;**107**:2183–90. doi:10.1111/j.1360-0443.2012.03972.x

50 Office for National Sstatistics. *The National Statistics Socio-economic Classification: User manual*. Basingstoke: Palgrave MacMillan 2005.

51 The National Institute for Health and Care Excellence. Guide to the methods of technology appraisal 2013. Published Online First: 2018.https://www.nice.org.uk/process/pmg9/resources/guide-to-the-methods-of-technology-appraisal-2013-pdf-2007975843781

52 Curtis L, Burns A. Unit Costs of Health and Social Care. Kent: 2019. doi:10.22024/UniKent/01.02.79286

53 Braun V, Clarke V. Using thematic analysis in psychology. *Qual Res Psychol* 2006;**3**:77–101.

54 Borland R., Partos TR, Yong HH, Cummings KM, Hyland A. How much unsuccessful quitting activity is going on among adult smokers? Data from the International Tobacco Control Four Country cohort survey. *Addiction* 2012;**107**:673–82. doi:10.1111/j.1360-0443.2011.03685.x

55 Abroms LC, Westmaas JL, Bontemps-Jones J, Ramani R, Mellerson J. A content analysis of popular smartphone apps for smoking cessation. *Am J Prev Med* 2008;**23**:1–7. doi:10.1038/jid.2014.371

56 Vilardaga R, Casellas-Pujol E, McClernon JF, *et al.* Mobile Applications for the Treatment of Tobacco Use and Dependence. *Curr Addict Reports* 2019;**6**:86–97. doi:10.1007/s40429-019-00248-0

57 McKay FH, Wright A, Shill J, *et al.* Using health and well-being apps for behavior

For peer review only - http://bmjopen.bmj.com/site/about/guidelines.xhtml

change: A systematic search and rating of apps. *JMIR mHealth uHealth* 2019;**7**:1–11. doi:10.2196/11926

58 Whittaker R, McRobbie H, Bullen C, *et al.* Mobile phone text messaging and app-based interventions for smoking cessation. *Cochrane Database Syst Rev* 2019;**2019**. doi:10.1002/14651858.CD006611.pub5

References from Tables

[1] Emery J, Coleman T, Sutton S, Cooper S, Leonardi-Bee J, Jones M, Naughton F. Uptake of Tailored Text Message Smoking Cessation Support in Pregnancy When Advertised on the Internet (MiQuit): Observational Study. *J Med Internet Res*. 2018;20(4):e146.

Figure 1 Flow diagram showing study design, measures and sample sizes

For peer review only - http://bmjopen.bmj.com/site/about/guidelines.xhtml

For peer review only

Figure 1 Flow diagram showing study design, measures and sample sizes

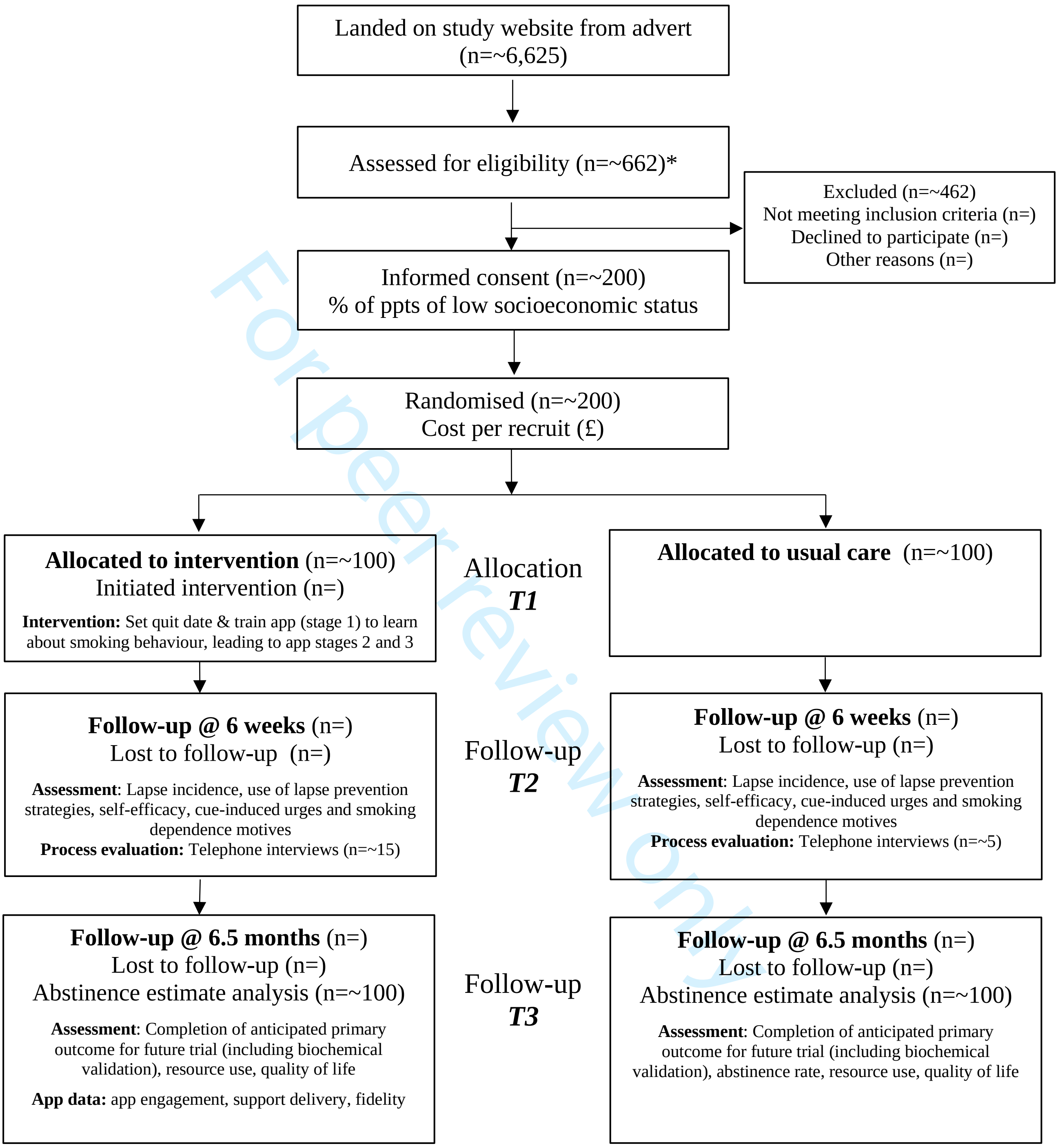

*Based on Emery et al (2018) [1] investigating the online uptake of a text message smoking cessation intervention. Approximately 10% of those visiting the landing page from paid-for Google and Facebook adverts completed eligibility questions, and of those 30% signed up.

For peer review only

Supplementary File

Protocol amendments.

Minor, non-notifiable, August 2020.

Minor, non-notifiable, December 2020.

Amendments to the Protocol and other documents (e.g. changes to eligibility criteria, outcomes, sample size calculations, analyses) will be agreed by the Trial Management Group (TMG). Such amendments will be forwarded to the Sponsor for confirmation as to whether it is either substantial or non-substantial and will then be submitted to the Ethics Committee for categorisation and approval prior to implementation.

Indemnity

University of East Anglia insurance shall apply.

Ancillary and post-trial care

The end of the trial is defined as 6 months following the last follow-up of the last participant randomised, to allow for data entry and data cleaning activities to be completed.

Once participants' involvement in the study has ended, they will no longer receive communications from the study team other than a copy of the findings, if they have requested one. Participants will be sent an SMS to inform them that their participation in the study has ended.

The NHS SmokeFree website support will continue be available after this study ends. Participants in the app group will still be able to access and use the Quit Sense app on their mobile if they wish to do so. If participants continue to use the app, it will continue to collect research data that we may use as part of the study analyses. After the study ends Quit Sense will no longer have technical support and participants will not be able to update or reinstall it. If participants wish, they can uninstall the app following the usual process for removing apps from their phone.

For peer review only - http://bmjopen.bmj.com/site/about/guidelines.xhtml

Data

Monitoring and management

The Chief Investigator has overall responsibility and will oversee study management. The Trial Management Group will be responsible for the running of the trial and will be supported by the Trial Steering Committee. A separate Data Monitoring and Ethics Committee is not considered necessary because the study is deemed to be low risk to participants.

Study data are collected and managed using REDcap (Research Electronic Data Capture). Data provided by trial participants will be entered under the participants PID number onto the central database stored on the servers based at the Norwich Clinical Trials Unit (NCTU). Access to the database will be via unique, individually assigned (i.e. not generic) usernames and passwords, and only accessible to members of the Quit Sense trial team and external regulators if requested. The servers are protected by firewalls and are patched and maintained according to best practice. The physical location of the servers is protected physically and environmentally in accordance with University of East Anglia's General Information Security Policy 3 (GISP3: Physical and environmental security).

The database and associated code have been developed by NCTU Data Management in conjunction with the Quit Sense trial team. The database software provides several features to help maintain data quality, including; maintaining an audit trail, allowing custom validations on all data, allowing users to raise data query requests, and search facilities to identify validation failure/ missing data. After completion of the trial the database will be retained on the servers of NCTU for on-going analysis of secondary outcomes.

Any transcriptions from the telephone interviews or app collected qualitative data will be anonymised. Voice recordings from telephone interviews and app recordings will be deleted after transcription, or if not transcribed then after audio coding for analysis.

Confidentiality and access to data

The investigators agree to archive and/or arrange for secure storage of Quit Sense trial materials and records for 20 years after the close of the trial unless otherwise advised by the NCTU. Personal contact details such as names, addresses and phone numbers will be destroyed 12 months after the end of the trial. Requests for access to trial data will be

For peer review only - http://bmjopen.bmj.com/site/about/guidelines.xhtml

considered, and approved in writing where appropriate, after formal application to the TMG or Trial Steering Committee (TSC). Considerations for approving access are documented in the TMG and TSC Terms of Reference. Location data will be kept for 20 years before being destroyed. This data will not be made available as an open data set outside of Quit Sense collaborations.

Concomitant Care

All participants will have access to treatment as usual for smoking cessation regardless of randomisation into this trial.

Roles and responsibilities

These membership lists are correct at the time of writing:

Protocol contributors

Name	Affiliation	Role
Felix Naughton	UEA	Led on protocol development
Juliet High	Norwich CTU	Contributed to regulatory and CTU related content
Aimie Hope	UEA	Contributed to protocol development
Caitlin Notley	UEA	Contributed to the qualitative process evaluation section
Lee Shepstone	Norwich CTU	Contributed to the statistical analysis section
Garry Barton	Norwich CTU	Contributed to the health economics section
Tim Coleman	University of Nottingham	Detailed comments on draft

Role of trial sponsor and funders

Name	Affiliation	Role
Helen Sutherland	South Norfolk CCG (‘Host’)	Representative of the host organisation
Graham Horne	UEA (‘Sponsor’)	Representative of the sponsor
Sheila Turner	NIHR	Representative of the funder

For peer review only - http://bmjopen.bmj.com/site/about/guidelines.xhtml

Trial Team

Name	Affiliation	Role and responsibilities
Felix Naughton	UEA	Chief investigator
Juliet High	Norwich CTU	Coordination of CTU contribution to project
Aimie Hope	UEA	Senior Research Associate; supporting trial delivery
Chloe Brown	University of Cambridge	Computer scientist leading app optimisation
Claire West	Norwich CTU	Development of study database and web-platform

Trial Management Group

Name	Affiliation	Role and responsibilities
Felix Naughton	UEA	Chief investigator
Juliet High	Norwich CTU	Coordination of CTU contribution to project
Aimie Hope	UEA	Senior Research Associate; supporting trial delivery
Tim Coleman	UEA	Co-applicant; mentoring support for CI
Caitlin Notley	UEA	Co-applicant; qualitative process evaluation advisor
Garry Barton	Norwich CTU	Co-applicant; health economics oversight
Chloe Brown	University of Cambridge	Computer scientist leading app optimisation
Ann Marie Swart	Norwich CTU	CTU oversight
Claire West	Norwich CTU	Development of study database and web-platform
Graham Horne	UEA	Representative of trial sponsor

Trial Steering Committee

Name	Affiliation	Role and responsibilities
Michael Ussher	St Georges/University of Stirling	Independent chair (clinical/behavioural science)
Aleksandra Herbec	UCL	Independent member (behavioural science)

For peer review only - http://bmjopen.bmj.com/site/about/guidelines.xhtml

Simon Coulton	University of Kent	Independent member (statistics/HE)
Qasim Chowdary	Public Health England	Independent member (services/clinical/policy)
Jo Hardy	PPI	Independent member (PPI)
Felix Naughton	UEA	Non-independent member (voting)
Juliet High	Norwich CTU	Observer
Helen Sutherland	South Norfolk CCG	Observer (host organisation)
Graham Horne	UEA	Observer (sponsor)
Aimie Hope	UEA	Observer

For peer review only

For peer review only - http://bmjopen.bmj.com/site/about/guidelines.xhtml

Appendix 2

Quit Sense Consent form

I understand that my participation is voluntary and that I am free to withdraw at any time, without giving any reason, and without my legal rights being affected.	
I understand that should I withdraw then the information collected so far cannot be erased and that this information may still be used in the project analysis.	
I understand that my personal details will be kept confidential.	
I understand that the data collected about me will be anonymised so that I am not identifiable, that study findings may be disseminated (e.g., in publications, academic conferences), and that fully anonymised data may be shared on academic open access platforms to ensure transparency, aid peer review and allow others to make use of important data for re-analysis.	
I understand and agree that I **may** be invited to verify my smoking status at the end of the study by giving a saliva sample by post, and that giving this sample is optional.	
I understand and agree that I **may** be invited to provide feedback on my experiences of this study and the digital support provided in a recorded telephone interview or by leaving a recorded message, and that giving my feedback is optional.	
I understand that if I provide feedback I may be quoted in published academic journals or presentations at public and academic events in the future, but to maintain my confidentiality I will not be named.	
I agree to my postal address being used in this study and consent to it being passed to the Quit Sense team. I understand my postal address will only be used for this study.	
I understand that **if** I am in the quit smoking app group, I will be offered access to a quit smoking app which will use my phone's in-built sensors (e.g. GPS) to record my location.	
I agree to my mobile phone number being used in this study and consent to it being passed to the Quit Sense team in case I will receive study related text messages. I understand my mobile number will only be used for this study.	
I agree to take part in the above study.	✓
Optional<br>Send me the findings.	✓

I agree to my contact details being held on a secure database until the end of the study so that I may receive a copy of<br>the results.
Name of participant<br><br>Date (entered automatically)

For peer review only

Reporting checklist for protocol of a clinical trial.

Based on the SPIRIT guidelines.

Instructions to authors

Complete this checklist by entering the page numbers from your manuscript where readers will find each of the items listed below.

Your article may not currently address all the items on the checklist. Please modify your text to include the missing information. If you are certain that an item does not apply, please write "n/a" and provide a short explanation.

Upload your completed checklist as an extra file when you submit to a journal.

In your methods section, say that you used the SPIRITreporting guidelines, and cite them as:

Chan A-W, Tetzlaff JM, Altman DG, Laupacis A, Gøtzsche PC, Krleža-Jerić K, Hróbjartsson A, Mann H, Dickersin K, Berlin J, Doré C, Parulekar W, Summerskill W, Groves T, Schulz K, Sox H, Rockhold FW, Rennie D, Moher D. SPIRIT 2013 Statement: Defining standard protocol items for clinical trials. Ann Intern Med. 2013;158(3):200-207

		Reporting Item	Page Number
**Administrative information**			
Title	#1	Descriptive title identifying the study design, population, interventions, and, if applicable, trial acronym	1

For peer review only

Trial registration	#2a	Trial identifier and registry name. If not yet registered, name of intended registry	2
Trial registration: data set	#2b	All items from the World Health Organization Trial Registration Data Set	
Protocol version	#3	Date and version identifier	19
Funding	#4	Sources and types of financial, material, and other support	19
Roles and responsibilities: contributorship	#5a	Names, affiliations, and roles of protocol contributors	1, 19
Roles and responsibilities: sponsor contact information	#5b	Name and contact information for the trial sponsor	20
Roles and responsibilities: sponsor and funder	#5c	Role of study sponsor and funders, if any, in study design; collection, management, analysis, and interpretation of data; writing of the report; and the decision to submit the report for publication, including whether they will have ultimate authority over any of these activities	20<br>Appendix 1 p. 3
Roles and responsibilities: committees	#5d	Composition, roles, and responsibilities of the coordinating centre, steering committee, endpoint adjudication committee, data management team, and	Appendix 1 p. 4-5

For peer review only

		other individuals or groups overseeing the trial, if applicable (see Item 21a for data monitoring committee)	
**Introduction**			
Background and rationale	#6a	Description of research question and justification for undertaking the trial, including summary of relevant studies (published and unpublished) examining benefits and harms for each intervention	4-5
Background and rationale: choice of comparators	#6b	Explanation for choice of comparators	9
Objectives	#7	Specific objectives or hypotheses	5
Trial design	#8	Description of trial design including type of trial (eg, parallel group, crossover, factorial, single group), allocation ratio, and framework (eg, superiority, equivalence, non-inferiority, exploratory)	5
**Methods: Participants, interventions, and outcomes**			
Study setting	#9	Description of study settings (eg, community clinic, academic hospital) and list of countries where data	7

For peer review only

		will be collected. Reference to where list of study sites can be obtained	
Eligibility criteria	#10	Inclusion and exclusion criteria for participants. If applicable, eligibility criteria for study centres and individuals who will perform the interventions (eg, surgeons, psychotherapists)	7
Interventions: description	#11a	Interventions for each group with sufficient detail to allow replication, including how and when they will be administered	9-12
Interventions: modifications	#11b	Criteria for discontinuing or modifying allocated interventions for a given trial participant (eg, drug dose change in response to harms, participant request, or improving / worsening disease)	9
Interventions: adherance	#11c	Strategies to improve adherence to intervention protocols, and any procedures for monitoring adherence (eg, drug tablet return; laboratory tests)	9
Interventions: concomitant care	#11d	Relevant concomitant care and interventions that are permitted or prohibited during the trial	Appendix 1 p. 3
Outcomes	#12	Primary, secondary, and other outcomes, including the specific measurement variable (eg, systolic blood pressure), analysis metric (eg, change from baseline, final value, time to event), method of aggregation (eg, median, proportion), and time point for each outcome. Explanation of the clinical relevance of chosen	12-14

For peer review only

		efficacy and harm outcomes is strongly recommended	
Participant timeline	#13	Time schedule of enrolment, interventions (including any run-ins and washouts), assessments, and visits for participants. A schematic diagram is highly recommended (see Figure)	Schedule pp. 6-7<br>Figure 1
Sample size	#14	Estimated number of participants needed to achieve study objectives and how it was determined, including clinical and statistical assumptions supporting any sample size calculations	14
Recruitment	#15	Strategies for achieving adequate participant enrolment to reach target sample size	8
**Methods: Assignment of interventions (for controlled trials)**			
Allocation: sequence generation	#16a	Method of generating the allocation sequence (eg, computer-generated random numbers), and list of any factors for stratification. To reduce predictability of a random sequence, details of any planned restriction (eg, blocking) should be provided in a separate document that is unavailable to those who enrol participants or assign interventions	14-15

For peer review only

Allocation concealment mechanism	#16b	Mechanism of implementing the allocation sequence (eg, central telephone; sequentially numbered, opaque, sealed envelopes), describing any steps to conceal the sequence until interventions are assigned	15
Allocation: implementation	#16c	Who will generate the allocation sequence, who will enrol participants, and who will assign participants to interventions	15
Blinding (masking)	#17a	Who will be blinded after assignment to interventions (eg, trial participants, care providers, outcome assessors, data analysts), and how	15
Blinding (masking): emergency unblinding	#17b	If blinded, circumstances under which unblinding is permissible, and procedure for revealing a participant's allocated intervention during the trial	N/A
**Methods: Data collection, management, and analysis**			
Data collection plan	#18a	Plans for assessment and collection of outcome, baseline, and other trial data, including any related processes to promote data quality (eg, duplicate measurements, training of assessors) and a description of study instruments (eg, questionnaires, laboratory tests) along with their reliability and	Schedule p. 6-7<br>Procedure p. 8-9

For peer review only

		validity, if known. Reference to where data collection forms can be found, if not in the protocol	Intervention (app) p. 10-12
Data collection plan: retention	#18b	Plans to promote participant retention and complete follow-up, including list of any outcome data to be collected for participants who discontinue or deviate from intervention protocols	9
Data management	#19	Plans for data entry, coding, security, and storage, including any related processes to promote data quality (eg, double data entry; range checks for data values). Reference to where details of data management procedures can be found, if not in the protocol	Appendix 1 p. 2-3
Statistics: outcomes	#20a	Statistical methods for analysing primary and secondary outcomes. Reference to where other details of the statistical analysis plan can be found, if not in the protocol	15-16
Statistics: additional analyses	#20b	Methods for any additional analyses (eg, subgroup and adjusted analyses)	16-17
Statistics: analysis population and missing data	#20c	Definition of analysis population relating to protocol non-adherence (eg, as randomised analysis), and any statistical methods to handle missing data (eg, multiple imputation)	15

For peer review only

Methods: Monitoring

Data monitoring: formal committee	#21a	Composition of data monitoring committee (DMC); summary of its role and reporting structure; statement of whether it is independent from the sponsor and competing interests; and reference to where further details about its charter can be found, if not in the protocol. Alternatively, an explanation of why a DMC is not needed	Appendix 1 p. 2-3
Data monitoring: interim analysis	#21b	Description of any interim analyses and stopping guidelines, including who will have access to these interim results and make the final decision to terminate the trial	N/A
Harms	#22	Plans for collecting, assessing, reporting, and managing solicited and spontaneously reported adverse events and other unintended effects of trial interventions or trial conduct	N/A
Auditing	#23	Frequency and procedures for auditing trial conduct, if any, and whether the process will be independent from investigators and the sponsor	N/A

Ethics and dissemination

Research ethics approval	#24	Plans for seeking research ethics committee / institutional review board (REC / IRB) approval	19

For peer review only

Protocol amendments	#25	Plans for communicating important protocol modifications (eg, changes to eligibility criteria, outcomes, analyses) to relevant parties (eg, investigators, REC / IRBs, trial participants, trial registries, journals, regulators)	Appendix 1 p. 1
Consent or assent	#26a	Who will obtain informed consent or assent from potential trial participants or authorised surrogates, and how (see Item 32)	8 Appendix 2
Consent or assent: ancillary studies	#26b	Additional consent provisions for collection and use of participant data and biological specimens in ancillary studies, if applicable	8
Confidentiality	#27	How personal information about potential and enrolled participants will be collected, shared, and maintained in order to protect confidentiality before, during, and after the trial	Appendix 1 p. 2-3
Declaration of interests	#28	Financial and other competing interests for principal investigators for the overall trial and each study site	19
Data access	#29	Statement of who will have access to the final trial dataset, and disclosure of contractual agreements that limit such access for investigators	Appendix 1 p. 2-3
Ancillary and post trial care	#30	Provisions, if any, for ancillary and post-trial care, and for compensation to those who suffer harm from trial participation	Appendix 1 p. 1

For peer review only

Dissemination policy: trial results	#31a	Plans for investigators and sponsor to communicate trial results to participants, healthcare professionals, the public, and other relevant groups (eg, via publication, reporting in results databases, or other data sharing arrangements), including any publication restrictions	2
Dissemination policy: authorship	#31b	Authorship eligibility guidelines and any intended use of professional writers	N/A
Dissemination policy: reproducible research	#31c	Plans, if any, for granting public access to the full protocol, participant-level dataset, and statistical code	See open data statement
**Appendices**			
Informed consent materials	#32	Model consent form and other related documentation given to participants and authorised surrogates	Appendix 2
Biological specimens	#33	Plans for collection, laboratory evaluation, and storage of biological specimens for genetic or molecular analysis in the current trial and for future use in ancillary studies, if applicable	9,13

None The SPIRIT checklist is distributed under the terms of the Creative Commons Attribution License CC-BY-ND 3.0. This checklist can be completed online using https://www.goodreports.org/, a tool made by the EQUATOR Network in collaboration with Penelope.ai

For peer review only

BMJ Open

Randomised controlled trial of a Just-In-Time Adaptive Intervention (JITAI) smoking cessation smartphone app: The Quit Sense feasibility trial protocol

Journal:	*BMJ Open*
Manuscript ID	bmjopen-2020-048204.R1
Article Type:	Protocol
Date Submitted by the Author:	06-Mar-2021

Complete List of Authors:	Naughton, Felix; University of East Anglia, Behavioural and Implementation Science Group, School of Health Sciences<br>Brown, Chloë; University of Cambridge, Department of Computer Science and Technology<br>High, Juliet; University of East Anglia, Norwich Clinical Trials Unit<br>Notley, Caitlin; University of East Anglia, Norwich Medical School<br>Mascolo, Cecilia; University of Cambridge, Department of Computer Science and Technology<br>Coleman, Tim; University of Nottingham, Division of General Practice<br>Barton, Garry; University of East Anglia, Norwich Medical School<br>Shepstone, Lee; University of East Anglia, Norwich Clinical Trials Unit<br>Sutton, Stephen; University of Cambridge, Behavioural Science Group<br>Prevost, A.; King's College London, Nightingale-Saunders Clinical Trials & Epidemiology Unit<br>Crane, David; University College London, Department of Behavioural Science and Health<br>Greaves, Felix; Imperial College London, Department of Primary Care and Public Health<br>Hope, Aimie; University of East Anglia, Behavioural and Implementation Science Group, School of Health Sciences

**Primary Subject Heading**:	Smoking and tobacco
Secondary Subject Heading:	Public health, Addiction
Keywords:	PUBLIC HEALTH, PREVENTIVE MEDICINE, QUALITATIVE RESEARCH

SCHOLARONE™
Manuscripts

I, the Submitting Author has the right to grant and does grant on behalf of all authors of the Work (as defined in the below author licence), an exclusive licence and/or a non-exclusive licence for contributions from authors who are: i) UK Crown employees; ii) where BMJ has agreed a CC-BY licence shall apply, and/or iii) in accordance with the terms applicable for US Federal Government officers or employees acting as part of their official duties; on a worldwide, perpetual, irrevocable, royalty-free basis to BMJ Publishing Group Ltd ("BMJ") its licensees and where the relevant Journal is co-owned by BMJ to the co-owners of the Journal, to publish the Work in this journal and any other BMJ products and to exploit all rights, as set out in our licence.

The Submitting Author accepts and understands that any supply made under these terms is made by BMJ to the Submitting Author unless you are acting as an employee on behalf of your employer or a postgraduate student of an affiliated institution which is paying any applicable article publishing charge ("APC") for Open Access articles. Where the Submitting Author wishes to make the Work available on an Open Access basis (and intends to pay the relevant APC), the terms of reuse of such Open Access shall be governed by a Creative Commons licence – details of these licences and which Creative Commons licence will apply to this Work are set out in our licence referred to above.

Other than as permitted in any relevant BMJ Author's Self Archiving Policies, I confirm this Work has not been accepted for publication elsewhere, is not being considered for publication elsewhere and does not duplicate material already published. I confirm all authors consent to publication of this Work and authorise the granting of this licence.

For peer review only

Title: Randomised controlled trial of a Just-In-Time Adaptive Intervention (JITAI) smoking cessation smartphone app: The Quit Sense feasibility trial protocol

Short title: Quit Sense feasibility trial protocol

Felix Naughton[1], Chloë Brown[2], Juliet High[3], Caitlin Notley[4], Cecilia Mascolo[2], Tim Coleman[5], Garry Barton[3,4], Lee Shepstone[3], Stephen Sutton[6], Toby Prevost[7], David Crane[8], Felix Greaves[9], Aimie Hope[1]

[1] Behavioural and Implementation Science Group, School of Health Sciences, University of East Anglia, UK
[2] Department of Computer Science and Technology, University of Cambridge, UK
[3] Norwich Clinical Trials Unit, University of East Anglia, UK
[4] Addiction Research Group, Norwich Medical School, University of East Anglia, UK
[5] Division of Primary Care, University of Nottingham, UK
[6] Behavioural Science Group, University of Cambridge, UK
[7] Nightingale-Saunders Clinical Trials & Epidemiology Unit, Kings College London, UK
[8] Department of Behavioural Science and Health, University College London, UK
[9] Department of Primary Care and Public Health, School of Public Health, Imperial College London, UK

* Corresponding author: Felix Naughton, Behavioural and Implementation Science Group, School of Health Sciences, University of East Anglia, Norwich, UK, NR4 7UL. Email: f.naughton@uea.ac.uk

Word count: 4,336

For peer review only - http://bmjopen.bmj.com/site/about/guidelines.xhtml

ABSTRACT

Introduction A lapse (any smoking) early in a smoking cessation attempt is strongly associated with reduced success. A substantial proportion of lapses are due to urges to smoke triggered by situational cues. Currently no available interventions proactively respond to such cues in real time. Quit Sense is a theory-guided Just-In-Time Adaptive Intervention (JITAI) smartphone app that uses a learning tool and smartphone sensing to provide in-the-moment tailored support to help smokers manage cue-induced urges to smoke. The primary aim of this randomised controlled trial (RCT) is to assess the feasibility of delivering a definitive online efficacy trial of Quit Sense.

Methods and analyses A two arm parallel-group RCT allocating smokers willing to make a quit attempt, recruited via online adverts, to usual care (referral to the NHS SmokeFree website) or usual care plus Quit Sense. Randomisation will be stratified by smoking rate (<16 vs. ≥ 16 cigarettes/day) and socioeconomic status (low vs. high). Recruitment, enrolment, baseline data collection, allocation and intervention delivery will be automated through the study website. Outcomes will be collected at 6 weeks and 6 months follow-up via the study website or telephone, and during app usage. The study aims to recruit 200 smokers to estimate key feasibility outcomes, the preliminary impact of Quit Sense, and potential cost-effectiveness, in addition to gaining insights on user views of the app through qualitative interviews.

Ethics and dissemination Ethics approval has been granted by the Wales NHS Research Ethics Committee 7 (19/WA/0361). The findings will be disseminated to the public, the funders, relevant practice and policy representatives and other researchers.

Trial registration: ISRCTN12326962.

Keywords: Smoking cessation, smartphone app, sensing, trial, online, just-in-time adaptive intervention

For peer review only - http://bmjopen.bmj.com/site/about/guidelines.xhtml

Article Summary

Strength and limitations of this study

- This is the first randomised controlled trial to evaluate a context-aware Just-In-Time Adaptive Intervention (JITAI) for smoking cessation.
- The study will identify key parameters to inform a definitive trial and will estimate intervention impact on biochemically validated abstinence from smoking.
- Recruitment, enrolment, baseline data collection, allocation, intervention delivery and most data collection during follow-up will be fully automated through the study website.
- There is potential for selection bias due to the requirement for participants to have internet access and an Android smartphone.

For peer review only

For peer review only - http://bmjopen.bmj.com/site/about/guidelines.xhtml

INTRODUCTION

Tobacco smoking is the second largest contributor of disease burden globally[1] and the largest in the UK.[2] While quitting reduces the risk of smoking related health problems and improves mental health,[3,4] the success rate remains low. Of the three million UK smokers attempting to quit annually, over 80% relapse within 12 months.[5]

Almost half of all lapses (any smoking) during a quit attempt are attributed to cravings triggered by environmental or situational cues[6] and lapses early in a quit attempt are highly predictive of longer-term relapse.[7–9] However, the most commonly used cessation medications do not alleviate cue-induced cravings.[10,11] Furthermore, while smokers are less likely to lapse if they use an effective cognitive and/or behavioural lapse prevention strategy[10,12,13] few do so.[12,14] There is a need for cessation interventions that effectively support the management of cue-induced cravings.

Unlike traditional cessation interventions smartphone apps have the advantage of portability and could therefore better assist with cue-induced cravings as they occur. Some mobile phone-based interventions have features specifically aimed at reducing lapse risk. Such support can be triggered by random Ecological Momentary Assessment (EMA) prompts[15] (where the smartphone prompts the user to complete assessment questions); or it can take the form of on-demand (i.e., user-initiated) requests for craving support.[16,17] However, given the rapid time to lapse after craving onset[10] and that many on-demand craving tools are seldom used beyond a first try,[17,18] these kinds of user-initiated features may still miss many craving episodes.

To address such limitations in current digital support we developed Quit Sense, a digital intervention which predicts and adapts to smoking risk in real time[19] (a Just-In-Time Adaptive Intervention). Quit Sense is a theory-guided smoking cessation tool that delivers ‘context aware’ lapse prevention support primarily using smartphone location sensing. It has demonstrated feasibility in delivering ‘in the moment’ support and acceptability among users.[20] We are now conducting a feasibility randomised controlled trial of Quit Sense to inform the design parameters of a future randomised control trial (RCT) and to provide estimates of intervention impact.

For peer review only

This trial is being conducted during the COVID-19 pandemic and as such different contextual variables may impact on study participants during their quit attempts (e.g., self-isolation, remote working) compared to before the COVID-19 pandemic. Through qualitative interviews and app recorded feedback this feasibility trial will capture participant views of the acceptability of the app and study, including how these are impacted by any COVID-19 restrictions.

Objectives

In this feasibility trial we aim to estimate:

a) Completion rates for the anticipated primary outcome for a full trial
b) Usual care arm cessation rate
c) Cost of recruitment using online advertising
d) Rates of app installation and use, and support delivery fidelity
e) Completion of smoking cessation-related resource use and quality of life data
f) Participant views of the app, as part of a qualitative process evaluation
g) Intervention effect on anticipated primary outcome
h) Intervention effect on hypothesised mechanisms of action of app at 6 weeks post-enrolment

METHODS AND ANALYSIS

Study design

A two-arm parallel-group randomised controlled feasibility trial, allocating smokers to either 'usual care' (link to NHS SmokeFree website)[21] or the intervention arm who receive 'usual care' plus access to the Quit Sense app.

Additionally, there is a nested qualitative process evaluation with a subsample of control and intervention participants to 1) assess intervention experiences and how these might help explain causal pathway(s) towards smoking behaviour change (intervention arm only)[22] and 2) asses experiences of study participation.

Additional information on study configuration can be found in Appendix 1.

For peer review only - http://bmjopen.bmj.com/site/about/guidelines.xhtml

Table 1 Schedule of participant assessments and intervention delivery

	STUDY PERIOD			
	Enrolment (online)	Allocation (online)	Post-allocation (online or telephone)	Close-out (online or telephone)
TIMEPOINT	0	0	*6 weeks FU (from enrolment)	**6 months FU (from enrolment)
ENROLMENT:				
Eligibility screen	X			
Informed consent	X			
Allocation		X		
INTERVENTIONS:				
Usual care (link to www.smokefree.nhs.uk)		X		
Quit Sense app + usual care		X		
ASSESSMENTS:				
Demographics	X			
Use of smartphone and apps	X			X
Smoking behaviour and dependence	X			X
Smoking beliefs	X			X
Cessation self-efficacy	X		X	X
Strength and frequency of urges to smoke	X		X	X

For peer review only - http://bmjopen.bmj.com/site/about/guidelines.xhtml

Automaticity and associative processes subscales of the WISDM-37	X		X	
EQ-5D-5L	X			X
Smoking lapse incidence			X	
Smoking abstinence outcomes			X	X
Use of lapse prevention strategies			X	
Smoking cessation resource use				X
Views on the app (Quit Sense arm only)				X
Assessment of Tobacco Exposure (postal saliva sample)				X

*Follow-up at 6 weeks (one month, plus 2 weeks to cover the likely quitting period/phase 1 of the app).

** Follow-up will be scheduled approximately 6 months post enrolment in the study, allowing for an additional 2 weeks to cover the likely quitting period (phase 1 of the app). Although in practice this will be 6 months plus 2 weeks, we will refer to it simply as the '6-month follow-up' for convenience.

Participants

Eligibility criteria

Participants will be smokers aged 16 years plus; currently smoke at least 7 cigarettes per week; willing to make a quit attempt within 14 days of enrolling; have primary use of an Android smartphone (version 5.0 and above) and will be resident in England. Participants must not have previously participated in this trial.

For peer review only - http://bmjopen.bmj.com/site/about/guidelines.xhtml

Study setting

Participants will complete screening, consent, baseline and follow-up measures online. This online design matches the ‘real world’ setting of smartphone app uptake.

Online recruitment

Recruitment is through paid-for online advertisements with Google Search and social media (Facebook and Instagram), limited to England-based IP addresses and targeted at Android devices. Advertisements are managed by ‘Nativve’,[23] specialists in digital marketing for research study recruitment. To maximise participation rate efficiency and in accordance with PPI recommendations, different adverts will be tested using A/B experiments while recruitment is live. People clicking on adverts will be directed to the study website and provided with study information including a downloadable ‘Participant Information Sheet.’ The screening and consent procedures are online with an e-signature confirming consent (Table 1, Appendix 2). After completing an online baseline questionnaire, participants will be randomly allocated to usual care or usual care plus Quit Sense. As described in Figure 1 and informed by a prior text message smoking cessation intervention,[24] we estimate that ~6,625 unique visitors are required to achieve a sample size of 200.

The distribution of socioeconomic status (SES) during recruitment will be monitored. Low SES is defined as individuals with a semi-routine or routine and manual occupation, class 5 in the National Statistics Socio-Economic Classification (NS-SEC),[25] or who have never worked or are long-term unemployed. Targeted advertising will be used to increase low SES representation if less than 45% of our sample are categorised as low SES when 35% of the target sample is reached.

Procedure

Both arms will automatically receive a link to NHS SmokeFree via SMS after enrolment. Only the Quit Sense arm will receive an SMS with an app link and activation code. Use of the activation code will be monitored. Participants who do not initiate the app will be sent an SMS reminder 3 days post enrolment and then five days post enrolment asking to identify a reason for not initiating the app from a predefined list.

For peer review only - http://bmjopen.bmj.com/site/about/guidelines.xhtml

To attempt to increase response rates all participants will receive an SMS prior to receiving links to follow-up questionnaires. Those who do not respond to the SMS with a survey link will be telephoned. Once the six-week questionnaire is completed, a subsample of participants from both arms (approximately 3:1 ratio between Quit Sense and usual care) will be invited to a telephone interview to gather data on their experiences of the app (intervention only) and of participating in the study. Where practicable we will use purposive sampling to interview a mixture of high and low SES abstainers and smokers, and for Quit Sense participants based on varying rates of app engagement. We anticipate interviewing 20 participants, depending on when data saturation is reached.

Six months and two weeks after enrolling (allowing for a two-week quitting preparation period for Quit Sense participants; henceforth referred to as the 'six-month' follow-up), all participants will be asked via SMS to complete the final questionnaire. Participants reporting abstinence in the previous 7 days at follow up will be posted a saliva sample kit to be returned to ABS laboratories, UK.

Participants will receive vouchers on completion of the 6-week (£5) and 6-month (£10) surveys, return of the saliva sample (£5) and for participating in an interview (£20).
As suggested by our PPI panel, all participants will receive a postcard and SMS to thank them and emphasise their importance to the study.

Criteria for discontinuing or modifying allocated interventions

Unless a participant withdraws from the study, they will be followed-up for data collection regardless of their level of engagement or smoking status. Participants who discontinue using the app for any reason other than withdrawal, will remain in the trial for the purpose of follow-up and data analysis.

For peer review only - http://bmjopen.bmj.com/site/about/guidelines.xhtml

Interventions

Usual Care Arm

Usual care is a web-link (NHS SmokeFree) sent by SMS. We consider this equivalent to 'usual care' for an online population. SmokeFree provides access and signposting to digital, telephone and in-person cessation support in England. At the time of writing the support offered includes information and links to six main types of support; support from a cessation advisor (via a Stop Smoking Service), the SmokeFree National telephone helpline, an online chat facility with an advisor, an email cessation programme, an SMS text message programme and the NHS SmokeFree smartphone app (28-day programme, non-tailored and not including proactive support delivery for managing urges to smoke).

Quit Sense app Arm

Quit Sense is a context-aware smartphone app designed for smokers wanting to quit. It is informed by learning theory (LT)[26,27] and two theory-guided SMS text message systems,[17,28] which are in turn informed by Social Cognitive Theory (SCT).[29] Quit Sense targets three key determinants that map onto LT and six mapping onto SCT: learned associations between smoking and an individual's physical environment (LT); learned associations between smoking and mood triggered by the environment (LT); the presence of other smokers (LT, SCT); awareness of smoking triggers (antecedents) (SCT); outcome expectancies of a lapse (SCT); goal/intention for complete abstinence (SCT); self-efficacy in resisting urges to smoke (SCT); knowledge of and self-efficacy for lapse prevention strategy use (SCT). Twenty-one corresponding Behaviour Change Techniques (BCTs)[30] are used to target these determinants.

The main feature is 'Geofence-Triggered Support' (GTS), which is orientated around three app stages:

- Stage 1 (training): The user sets a quit date (default suggestion 7 days) and trains the app to learn about their smoking behaviour. This requires the user to report in the app each smoking episode and the situational context in real time (stress, mood, urge strength, setting description [home, working, home-working, socialising, other], presence of other smokers), while the app records geolocation. If a user reports smoking more than once in the same location (as defined by the app) a geofence (a

For peer review only
For peer review only - http://bmjopen.bmj.com/site/about/guidelines.xhtml

circular virtual perimeter) is created representing a high-risk area. Location monitoring precision varies depending on the availability of GPS/WiFi/network (range ~ 5-50 m).

- Stage 2 (28-day abstinence challenge): Post quit date, the app monitors the user's location. A support message is triggered a minimum of 1 minute after the user enters a geofence zone (typical range 1-15 mins, depending on operating system). Messages are individually tailored using Stage 1 context information and using any stage 2 smoking reports, providing support to help users avoid or cope with location specific triggers. Further messages are triggered after each 3-hour interval of remaining in that location (default between 8am-9.30pm or as defined by the user). This also applies to 'home-working' messages. However, if the user has not reported 'home-working' within the time window (2-3 hours) in which the message is to be delivered, a 'home' message will be delivered instead. This is because 'time' of reporting is an additional requirement (to 'location') for home-working messages to be triggered.
- Stage 3 (maintaining abstinence): The app continues to deliver GTS for 2 further months but reduces the frequency by one-half every month. After 3 months post-quit date, the GTS support stops, unless the user opts to switch it back on or restart their quit attempt.

Quit Sense has six additional features (relevant to all stages, unless specified otherwise):

1. Feedback is provided after each smoking report. In Stage 1 this is tailored to nine characteristics collected during app initiation. Messages focus on enhancing quitting preparation, motivation and self-efficacy. In Stage 2, feedback is non-tailored and orientated around lapse and relapse prevention.
2. An 'End of Day' (EoD) survey that users are prompted to complete, recording cigarettes smoked that day, cravings[31] and self-efficacy[28,29] (Stages 1 and 2 only). Feedback is provided after each EoD survey, including encouragement to log smoking behaviour if there is a discrepancy between the survey and real time reports.
3. A 'my profile' screen including number of days quit, money saved (based on smoking rate and reported cost), a calendar showing a heat map and 'smileys' (use of yellow, grey and blue to denote values with corresponding happy, neutral and sad smileys) for smoking, cravings and self-efficacy for each EoD survey

For peer review only - http://bmjopen.bmj.com/site/about/guidelines.xhtml

completed. In addition, smoking pattern feedback is provided using graphical and written summaries for smoking triggers based on the user's historical reporting.

4. A 'library' of quitting advice split into six categories: 'getting ready', 'boost your motivation', effects of smoking and quitting', 'early days of quitting', 'staying quit', 'smoking after your quit date'. Users can write and submit their own support messages.
5. Scheduled non-tailored daily support messages delivered each morning orientated around the quit date (Stages 1 and 2 only) – targeting outcome expectancies, motivation, preparation, self-efficacy (three sets of different messages to prevent message duplication in successive attempts).
6. The option for the user to reset their quit date in Stage 1, if they are not feeling ready, or Stage 2 if they relapse. Relapse is defined as multiple smoking episodes that are reported over two days during a quit attempt (either through smoking episode logs or the EoD survey). If this occurs, users are invited to either re-commit and continue or reset their quit date and return to Stage 1.

Qualitative process evaluation

The aims of the qualitative process evaluation are:

1. To gather user experiences of app usage in order to inform app optimisation.
2. To understand participant experiences of participation and the unique contexts in which participants are attempting to quit.

Interview questions will probe for in-depth descriptions of intervention participants' experiences of app usage including which types of support they liked most and least, the timing of support in relation to experienced need for support, and views on the features used. Control participants will be asked about their experience of trial participation. Both groups will be asked about their use of or interest in other smoking cessation aids and support and contextual details such as mandated movement restrictions and family setting. In addition, the Quit Sense app includes an in-built audio record feature so that users can leave feedback. The study will therefore also assess the feasibility of a user-initiated process evaluation approach where data is collected with high ecologically validity. Any relevant data submitted, will be analysed alongside interview data, potentially providing a broader range of views from a more user-controlled perspective. Transcriptions from interviews or app recordings will be

For peer review only - http://bmjopen.bmj.com/site/about/guidelines.xhtml

For peer review only

anonymised. Recordings will be deleted after transcription, or if not fully transcribed, after audio coding for analysis.

Database

Study data are collected and managed using REDcap (Research Electronic Data Capture) tools hosted at Norwich Clinical Trials Unit (NCTU). REDcap is a secure, web-based software platform supporting data capture for research studies.[32,33] Further details on data management can be found in the online Supplementary File.

Outcomes

Feasibility Outcomes

As this is a feasibility trial there is no primary outcome. Feasibility outcomes will be used to estimate key parameters to inform a future trial,[34] and include:

i. Completeness of the anticipated primary outcome for a future definitive trial (6-month self-reported abstinence with biochemical validation; see smoking outcomes section).
ii. Abstinence rate of usual care arm, using the anticipated primary outcome for a future definitive trial.
iii. Cost per recruit, based on advertising costs.
iv. Rates of app installation use and support delivery fidelity.
v. Completion of smoking cessation-related resource use, including usual care use, and quality of life (EQ-5D-5L)[35] data.
vi. Hypothesised mechanisms of action of Quit Sense.
vii. Participant experiences and feedback on app usage.

Smoking and related outcomes

Smoking and related outcomes will provide preliminary information about the intervention's impact. The main abstinence outcome for which we will monitor completeness of ascertainment is based on the Russell standard.[36] Abstinence will be defined as: self-reported abstinence in the previous 6 months allowing for no more than five cigarettes and not smoking in the previous week, biochemically validated by a saliva cotinine concentration of less than 10 ng/ml[36,37] and for those using nicotine substitution (e.g. Nicotine

For peer review only - http://bmjopen.bmj.com/site/about/guidelines.xhtml

Replacement Therapy, or e-cigarettes) an anabasine concentration of less than 0.2 ng/ml , based on feedback from our testing laboratory.[37,38] Thresholds will be reviewed prior to analysis in case of changes in guidance or relevant evidence. Given recent evidence that some e-liquid, believed to be that made outside of the UK, can contain anabasine,[37] we will monitor the proportion of e-cigarette users who report abstinence from smoking but have an anabasine level above the chosen threshold.

Smoking cessation experts highlight the value of additional smoking outcomes based on different time periods of abstinence and time-points.[39] We will therefore measure 7-day point prevalence abstinence at 6 months follow-up (self-report and biochemically verified) and 7-day point prevalence abstinence at 6 weeks follow-up (self-report).

We will collect the following hypothesised mechanisms of action at 6-week follow-up:

- Lapse incidence (any smoking, even a puff) since the initial quit attempt (if made),[6] which is highly predictive of relapse.[7–9]
- Lapse prevention strategy use,[14] which is associated with lapse prevention.[10]
- Self-efficacy,[28,29] which prospectively predicts lapse and relapse to smoking.[40]
- The Strength of Urges to Smoke (SUTS) measure,[31] which prospectively predicts abstinence and is superior to other urge-measures in doing so,[41] and Frequency of Urges to Smoke (FUTS).[41]
- Automaticity and associative processes subscales from the Wisconsin Inventory of Smoking Dependence Motives (WISDM-37).[42]

Sample size

In line with recommendations that feasibility studies with binary outcome measures recruit at least 60 participants per group (minimum of 120) and a maximum of 100 per group, the proposed sample size for this trial is 200 smokers (100 per arm).[43] Participants will be randomised to usual care or Quit Sense arms on a 1:1 ratio. This sample size will enable key parameters to inform a future definitive trial to be estimable within the following precision (defined as the 95% confidence interval [CI] half-width):

- Primary outcome completion: we estimate a follow-up rate for self-reported smoking status at 6 months of 80%, with precision of +/- 6%.[44–46] For

For peer review only - http://bmjopen.bmj.com/site/about/guidelines.xhtml

biochemical validation, we estimate that 75% of participants reporting abstinence will return a saliva sample by post, with precision of +/- 22%.[28,45]

- Cessation rate in usual care arm: we estimate an abstinence rate of 5% at 6 months follow-up, providing precision of +/- 4%.[47]
- App installation and initiation: we estimate that 85% of intervention participants will install the app, with precision of +/- 7%.[48]
- App engagement – in our acceptability study, 71% used the app for more than one week (a timeframe deemed meaningful by our PPI panel). With a similar rate in the trial, precision would be +/- 9%. This is in line with other studies.[48]

Randomisation

Sequence generation, allocation and blinding

Randomisation will be stratified by smoking rate (<16 vs. ≥ 16 cigarettes/day; based on mean smoking rates from trials recruiting smokers online)[45,49] and socioeconomic status (low vs. high), based on the National Statistics Socio Economic Classification (NS-SEC) self-coded method.[50] Allocation sequences will be generated by a computer-based random number generator using random permuted blocks (varying block sizes) (using REDcap). Randomisation will be integrated into the enrolment process on the study website. The system will not permit a participant to enrol twice with the same mobile number or email address. As allocation will be integrated into the study website, the allocation sequence will be concealed from participants until assignment and concealed from members of the trial team, except the statistician, developers of the study database and the research associate supporting trial delivery.

Data analysis

The feasibility and acceptability of all measures will be assessed by their level of completeness and via interviews and app audio data.

For peer review only - http://bmjopen.bmj.com/site/about/guidelines.xhtml

For peer review only

Outcomes

Completeness of the anticipated primary outcome for a future trial (outcome i.) and the abstinence rate using this anticipated primary outcome for the usual care arm (outcome ii.) will be described as proportions with 95% CIs, and translated into interpretable probabilities using the Bayesian approach relevant in preliminary trials with the objective of powering the definitive trial.

Cost per recruit (outcome iii.), rates of app installation (outcome iv.) and completion of smoking cessation-related resource use and quality of life (EQ-5D-5L) (outcome v.) will be described using summary statistics with 95% CIs.

We will estimate the preliminary intervention effect on abstinence, lapse incidence and use of lapse prevention strategies, using multiple logistic regression, providing odds ratios with 95% CIs, while adjusting for any potential baseline confounders defined by the Statistical Analysis Plan (SAP). For the estimated intervention effect on abstinence we will assume missing=smoking.[36] To estimate intervention effects on self-efficacy, urges and smoking dependence motives, we will use ANCOVA models involving adjustment for the baseline score and for pre-defined potential confounders, primarily using complete cases only. Missing outcome assumptions will be assessed through sensitivity analyses which will be defined in the SAP.

Economic evaluation

We aim to collect data (baseline and 6-months follow-up) that will enable us to inform the decision as to how cost (based on resource use data) and benefit (in terms of quality of life [QoL]) data are estimated in any future more definitive study. Completion rates will thereby be estimated.

In terms of costs, we will estimate those associated with the intervention (e.g. app maintenance) and smoking related costs to both the individual (e.g. nicotine replacement therapy) and the NHS (e.g. NHS stop smoking services/GP visits). In line with NICE guidance,[51] QoL will be measured via the EQ-5D-5L[35] as this enables the calculation of

For peer review only - http://bmjopen.bmj.com/site/about/guidelines.xhtml

QALY (Quality Adjusted Life Year) scores. Appropriate unit costs (e.g.[52]) will also be attached to all items of resource-use in order to identify major cost drivers.

Based on the intention to treat approach, a preliminary cost-effectiveness analysis will be performed. In the base-case analysis, costs will be estimated from the viewpoint of the NHS and personal social services.[51] We will estimate the mean incremental cost and mean QALY gain associated with Quit Sense compared to usual care. Assuming dominance does not occur (where one intervention is both more costly and less effective), then the incremental cost effectiveness ratio (incremental cost/QALY gain) will be estimated and assessed in relation to a range of cost-effectiveness thresholds (e.g. £20,000 to £30,000 per QALY),[51] in order to provide a preliminary assessment of whether Quit Sense constitutes value for money. The associated level of uncertainty will also be estimated.

Qualitative process evaluation data collection and analysis

Interviews will be audio-recorded and transcribed intelligent verbatim. App audio-recordings may be transcribed depending on quality and resources. If transcription is unfeasible, coding of audio files will be completed.

We will undertake an inductive thematic analysis of the first 3-4 interview transcripts,[53] develop a coding framework from this, and then code remaining data. We will check coding consistency through the independent coding of 10% of the data, with a further 10% if consistency is unsatisfactory. We will continue refining until the themes are agreed. Analysis of process evaluation data will focus on identifying key themes associated with using the app and views and experiences of using other relevant apps. A second stage of analysis will work with descriptions of experiences of lapse or lapse avoidance in the context of the app and considering unique participant contexts. These will be reported as vignettes and used to explore participant experiences and to identify mechanisms of action and casual pathways towards behaviour change.

Patient and Public Involvement

We have a patient and public involvement (PPI) panel of 4 former smokers who have contributed to study design including the recruitment strategy, wording of SMS messages,

For peer review only - http://bmjopen.bmj.com/site/about/guidelines.xhtml

For peer review only

study information, the qualitative evaluation, the design of the study logo, and the function and features of the Quit Sense app and support it delivers. The PPI panel is represented on the Trial Steering Committee.

DISCUSSION

A feasibility trial of Quit Sense will identify the conditions necessary for a definitive trial, including for successful study design and data collection. This will largely be determined by whether: the recruitment strategy is able to produce the required sample size and participant retention rates; the rate of app installation; completion rates for follow-up and the anticipated primary outcome for a full trial; and participant views of the acceptability of the app and study. A potential future RCT will enable us to establish the extent to which the Quit Sense app is effective and cost effective relative to the usual provision of online stop smoking support.

This remotely delivered and largely automated trial is being conducted during the COVID-19 pandemic, a time when health protection measures are restricting or preventing many everyday behaviours. As such, there are likely to be different contextual variables impacting on study participants (e.g., self-isolation, remote working, limited opportunities to socialise, etc.) compared to before the pandemic. The Quit Sense app can learn about such situational factors because of the initial training phase where the user reports on their smoking behaviour. Furthermore, we hope to capture in-depth insights into the unique contexts in which participants are attempting to make their quit attempts through telephone interviews and the app's inbuilt audio-record feature.

The potential of intelligent and 'always on' digital cessation tools in providing on-hand effective and low cost support means that they are likely to be of growing importance in enhancing chances of successful quitting.[54] However, measures are needed to realise the potential of next generation digital cessation interventions most of which remain untested.[15] Where app evaluations have been undertaken results show that evidence-based smoking cessation strategies are underutilized,[55] that many use a limited number of behaviour change techniques,[56,57] and that few utilise features such as geolocation or just-in-time sensors.[56] It is crucial, therefore, that their effectiveness and cost effectiveness are established, particularly through use of randomised control trials.[58]

For peer review only - http://bmjopen.bmj.com/site/about/guidelines.xhtml

For peer review only

Quit Sense has been developed to address many of the current limitations in digital support. The app is distinct from other cessation support available due to its ability to identify location-based high-risk locations using smartphone sensing to provide timely theory-guided and tailored 'in-the-moment' support. Quit Sense has been developed to help smokers avoid or manage urges to smoke triggered by cues within these high-risk locations. This is done through the provision of lapse prevention strategies, outcome expectancy management and motivational and self-efficacy enhancement. Ultimately this support aims to help extinguish learned associations between smoking and a person's physical, social and psychological environment in order to reduce future relapse risk.

ETHICS AND DISSEMINATION

Ethics approval has been granted by the Wales NHS Research Ethics Committee 7 (19/WA/0361). The findings will be disseminated to the public, the funders, relevant practice and policy representatives and other researchers. A fully anonymised dataset from the trial will be made available upon reasonable request.

For peer review only - http://bmjopen.bmj.com/site/about/guidelines.xhtml

Author affiliations

University of East Anglia

Norwich Clinical Trials Unit

University of Nottingham

Author Contributions

FN, CN, CM, TC, GB, SS, TP, DC and FG conceived the study project and obtained project funding. FN led on app design, CB led on app development and AH and CB contributed to app design. FN and AH led on protocol development and drafted the manuscript.
CN contributed to the qualitative process evaluation section. GB led on the health economics section. LS contributed to the statistical analysis section. JH contributed to regulatory and Clinical Trials Unit related content. All authors contributed to the preparation and drafting of the manuscript.

Funding

This study is funded by the National Institute for Health Research (NIHR) Public Health Research Programme (17/92/31). Tim Coleman is an NIHR Senior Investigator.

Disclaimer

The views expressed are those of the authors and not necessarily those of the NIHR or the Department of Health and Social Care.

Competing interests

FG is also employed by NICE. DC is founder and CEO of a smoking cessation app called Smoke Free (23 Limited), which has no connection to Quit Sense.

Patient consent for publication

Not required.

Ethics approval

Ethical approval for this study was granted on the 28th of January 2020 (Wales REC 7, reference 19/WA/0361, IRAS project ID 270432).

Providence and peer review

Not commissioned, peer reviewed for ethical and funding approval prior to submission.

For peer review only - http://bmjopen.bmj.com/site/about/guidelines.xhtml

For peer review only

Open access

There is no data associated with this manuscript.

Protocol version 1.3 Date 15/12/2020

Trial sponsor

Graham Horne,

Research and Innovation Services,

University of East Anglia,

Norwich Research Park,

NR4 7TJ

research.sponsor@uea.ac.uk

01603 591477

Trial status

Recruiting

For peer review only

For peer review only - http://bmjopen.bmj.com/site/about/guidelines.xhtml

References

1 Roser M, Ritchie H. Burden of Disease. 2016. https://ourworldindata.org/burden-of-disease (accessed 27 Apr 2020).

2 Murray CJ, Richards MA, Newton JN, Fenton KA, Anderson HR, Atkinson C, Bennett D, Bernabé E, Blencowe H, Bourne R, Braithwaite T, Brayne C, Bruce NG, Brugha TS, Burney P, Dherani M, Dolk H, Edmond K, Ezzati M, Flaxman AD, Fleming TD, Freedman G, Gunnell D DA. UK health performance: findings of the Global Burden of Disease Study 2010. *Lancet* 2013;**381**:997–1020. doi:10.1016/S0140-6736(13)60355-4

3 National Center for Chronic Disease Prevention and Health Promotion (US) Office on Smoking and Health. The Health Consequences of Smoking - 50 years of Progress: A report of the Surgeon General. 2014. https://www.ncbi.nlm.nih.gov/books/NBK179276/

4 Taylor G, McNeill A, Girling A, Farley A, Lindson-Hawley N AP. Change in mental health after smoking cessation: Systematic review and meta-analysis. *BMJ* 2014;**348**:2216. doi:10.1136/bmj.g2216

5 West, R, Brown J. Smoking Toolkit Study. 2017. http://www.smokinginengland.info/ (accessed 27 Apr 2020).

6 Shiffman S, Paty JA, Gnys M, Kassel JA, Hickcox M. First lapses to smoking: within-subjects analysis of real-time reports. *J Consult Clin Psychol* 1996;**64**:366-379. doi:10.1037//0022-006x.64.2.366

7 Kenford SL, Fiore MC, Jorenby DE, Smith SS, Wetter D, Baker TB. Predicting Smoking Cessation. Who Will Quit With and Without the Nicotine Patch. *JAMA* 1994;**8**:589-94.https://www.ncbi.nlm.nih.gov/pubmed/8301790

8 Deiches DF, Baker TB, Lanza S, Piper ME. Early Lapses in a Cessation Attempt: Lapse Contexts, Cessation Success, and Predictors of Early Lapse. *Nicotine Tob Res* 2013;**15**:1883–91. doi:10.1093/ntr/ntt074

9 Shadel WG, Martino SC, Setodji C, Cervone D, Witkiewitz K, Beckjord EB, Scharf D, Shih R. Lapse-induced surges in craving influence relapse in adult smokers: an experimental investigation. *Health Psychol* 2011;**30**:588–96.

10 Ferguson SG, Shiffman S. The relevance and treatment of cue-induced cravings in tobacco dependence. *J Subst Abuse Treat* 2009;**36**:235–43. doi:10.1016/j.jsat.2008.06.005

For peer review only - http://bmjopen.bmj.com/site/about/guidelines.xhtml

11 Ferguson SG, Shiffman S. Effect of high-dose nicotine patch on craving and negative affect leading up to lapse episodes. *Psychopharmacology (Berl)* 2014;**231**:2595–602. doi:10.1007/s00213-013-3429-6

12 Brodbeck J, Bachmann MS, Znoj H. Distinct Coping Strategies Differentially Predict Urge Levels and Lapses in a Smoking Cessation Attempt. *Addict Behav* 2013;**38**:2224–9. doi:10.1016/j.addbeh.2013.02.001

13 O'Connell KA, Hosein VL, Schwartz JE. Thinking and/or doing as strategies for resisting smoking. *Res Nurs Heal* 2006;**29**:533–42. doi:10.1002/nur.20151

14 Naughton F, McEwen A, Sutton S. Use and effectiveness of lapse prevention strategies among pregnant smokers. *J Health Psychol* 2015;**20**:1427–33. doi:10.1177/1359105313512878

15 Hébert ET, Ra CK, Alexander AC, *et al.* A Mobile Just-in-Time Adaptive Intervention for Smoking Cessation: Pilot Randomized Controlled Trial. *J Med Internet Res* 2020;**22**:e16907. doi:10.2196/16907

16 Free C, Knight R, Robertson S, *et al.* Smoking cessation support delivered via mobile phone text messaging (txt2stop): A single-blind, randomised trial. *Lancet* 2011;**378**:49–55. doi:10.1016/S0140-6736(11)60701-0

17 Naughton F, Jamison J, Boase S, *et al.* Randomized controlled trial to assess the short-term effectiveness of tailored web- and text-based facilitation of smoking cessation in primary care (iQuit in Practice). *Addiction* 2014;**109**:1184–93. doi:10.1111/add.12556

18 Devries KM, Kenward MG, Free CJ. Preventing Smoking Relapse Using Text Messages: Analysis of Data From the txt2stop Trial. *Nicotine Tob Res* 2012;**15**:77–82. doi:https://doi.org/10.1093/ntr/nts086

19 Naughton F. Delivering 'Just-in-Time' smoking cessation support via mobile phones: Current knowledge and future directions. *Nicotine Tob Res* 2016;**19**:379–83 doi: 10.1093/ntr/ntw143

20 Naughton F, Hopewell S, Lathia N, *et al.* A Context-Sensing Mobile Phone App (Q Sense) for Smoking Cessation: A Mixed-Methods Study. *JMIR mHealth uHealth* 2016;**4**:e106. doi:10.2196/mhealth.5787

21 NHS Digital. SmokeFree. Online. 2020 https://www.nhs.uk/smokefree

22 Moore GF, Audrey S, Barker M, *et al.* Process evaluation of complex interventions: Medical Research Council guidance. *BMJ* 2015;**350**:1–7. doi:10.1136/bmj.h1258

23 Nativve: Digital Marketing Specialists. 2020. https://www.nativve.com/

24 Emery JL, Coleman T, Sutton S, Cooper S, Leonardi-Bee J, Jones M, Naughton F.

For peer review only - http://bmjopen.bmj.com/site/about/guidelines.xhtml

Uptake of Tailored Text Message Smoking Cessation Support in Pregnancy When Advertised on the Internet (MiQuit): Observational Study. *J Med Internet Res* 2018;**20**:e146. doi:10.2196/jmir.8525.

25 Office for National Statistics. The National Statistics Socio-economic classification (NS-SEC). Online. 2016. https://www.ons.gov.uk/methodology/classificationsandstandards/otherclassifications/thenationalstatisticssocioeconomicclassificationnssecrebasedonsoc2010

26 Pavlov I. *Conditioned reflexes*. London and New York: Oxford University Press 1927.

27 Skinner B. *The behavior of organisams: An experimental analysis*. New York: Appleton-Century 1938.

28 Naughton F, Prevost AT, Gilbert H, Sutton S. Randomised controlled trial evaluation of a tailored leaflet and SMS text message self-help intervention for pregnant smokers. *Nicotine Tob Res* 2012;**14**:569–77. doi::10.1093/ntr/ntr254

29 Bandura A. *Social foundations of thought and action: A social cognitive theory*. Englewood Cliffs: Prentice-Hall 1986.

30 Michie S, Richardson M, Johnston M, Abraham C, Francis J, Hardeman W, Eccles MP, Cane J, Wood CE. The behavior change technique taxonomy (v1) of 93 hierarchically clustered techniques: building an international consensus for the reporting of behavior change interventions. *Ann Behav Med* 2013;**46**:81–95. doi:10.1007/s12160-013-9486-6

31 West R, Hajek P. Evaluation of the Mood and Physical Symptoms Scale (MPSS) to Assess Cigarette Withdrawal. *Psychopharmacol* 2004;**177**:195–9. doi:10.1007/s00213-004-1923-6

32 Harris PA, Taylor R, Thielke R, Payne J, Gonzalez N, Conde JG. Research electronic data capture (REDCap)--a metadata-driven methodology and workflow process for providing translational research informatics support. *J Biomed Inf* 2009;**42**:377–81. doi:10.1016/j.jbi.2008.08.010

33 Harris PA, Taylor R, Minor BL, Elliott V, Fernandez M, O'Neal L, McLeod L, Delacqua G, Delacqua F, Kirby J, Duda SN, REDCap Consortium. The REDCap consortium: Building an international community of software platform partners. *J Biomed Inform* 2019;**95**. doi:10.1016/j.jbi.2019.103208.

34 Craig P, Dieppe P, Macintyre S, *et al.* Developing and evaluating complex interventions: The new Medical Research Council guidance. *BMJ* 2008;**337**:979–83. doi:10.1136/bmj.a1655

For peer review only - http://bmjopen.bmj.com/site/about/guidelines.xhtml

35 Herdman M, Gudex C, Lloyd A, *et al.* Development and preliminary testing of the new five-level version of EQ-5D (EQ-5D-5L). *Qual Life Res* 2011;**20**:1727–36. doi:10.1007/s11136-011-9903-x

36 West R, Hajek P, Stead L, Stapleton J. Outcome Criteria in Smoking Cessation Trials: Proposal for a Common Standard. *Addiction* 2005;**100**:299–303. doi:10.1111/j.1360-0443.2004.00995.x.

37 Benowitz NL, Bernert JT, Foulds J, Hecht SS, Jacob P, Jarvis MJ, Joseph A, Oncken C, Piper ME. Biochemical Verification of Tobacco Use and Abstinence: 2019 Update. *Nicotine Tob Res* doi:10.1093/ntr/ntz132

38 Jacob P, Hatsukami D, Severson H, Hall S, Yu L, Benowitz N. Anabasine and anatabine as biomarkers for tobacco use during nicotine replacement therapy. *Cancer Epidemiol Biomarkers Prev* 2002;**11**:1668–73.

39 Cheung KL, De Ruijter D, Hiligsmann M, *et al.* Exploring consensus on how to measure smoking cessation. A Delphi study. *BMC Public Health* 2017;**17**:1–10. doi:10.1186/s12889-017-4902-7

40 Gwaltney CJ, Shiffman S, Balabanis MH, Paty JA. Dynamic self-efficacy and outcome expectancies: prediction of smoking lapse and relapse. *J Abnorm Psychol* 2005;**114**:661–75. doi:10.1037/0021-843X.114.4.661

41 Fidler JA, Shahab LS, West R. Strength of urges to smoke as a measure of severity of cigarette dependence: Comparison with the Fagerstrom Test for Nicotine Dependence and its components. *Addiction* 2010;**106**:631–8. doi:10.1111/j.1360-0443.2010.03226.x

42 Smith SS, Piper ME, Bolt DM, Fiore MC, Wetter DW, Cinciripini PM, Baker TB. Development of the Brief Wisconsin Inventory of Smoking Dependence Motives. *Nicotine Tob Res* 2010;**12**:489–99. doi:10.1093/ntr/ntq032

43 Teare MD, Dimairo M, Shephard N, *et al.* Sample size requirements to estimate key design parameters from external pilot randomised controlled trials: a simulation study. *Trials* 2014;**15**:264.

44 Bricker JB, Mull KE, Kientz JA. Randomized, controlled pilot trial of a smartphone app for smoking cessation using acceptance and commitment therapy. *Drug Alcohol Depend* 2014;**143**:87–94. doi:doi:10.1016/j.drugalcdep.2014.07.006.

45 Brown J, Michie S, Geraghty AWA, Yardley L, Gardner B, Shahab L, Stapleton JA, West R. Internet-based intervention for smoking cessation (StopAdvisor) in people with low and high socioeconomic status: a randomised controlled trial. *Lancet Respir*

For peer review only - http://bmjopen.bmj.com/site/about/guidelines.xhtml

Med 2014;**2**:997–1006. doi:10.1016/S2213-2600(14)70195-X

46 Taylor GMJ, Dalili MN, Semwal M, Civljak M, Sheikh A, Car J. Internet-based interventions for smoking cessation. Cochrane Database Syst Rev. *Cochrane Database Syst Rev* 2017;9(9) doi:10.1002/14651858.CD007078.pub5

47 Whittaker R, Mcrobbie H, Bullen C, Rodgers A, Gu Y. Mobile phone-based interventions for smoking cessation. *Cochrane Database Syst Rev* 2016; 10;4(4). doi:10.1002/14651858.CD006611.pub4

48 Iacoviello BM, Steinerman JR, Klein DB, *et al.* Clickotine, A Personalized Smartphone App for Smoking Cessation: Initial Evaluation. *JMIR mHealth uHealth* 2017;**5**:e56. doi:10.2196/mhealth.7226

49 Mason D, Gilbert H, Sutton S. Effectiveness of web-based tailored smoking cessation advice reports (iQuit): A randomized trial. *Addiction* 2012;**107**:2183–90. doi:10.1111/j.1360-0443.2012.03972.x

50 Office for National Sstatistics. *The National Statistics Socio-economic Classification: User manual*. Basingstoke: Palgrave MacMillan 2005.

51 The National Institute for Health and Care Excellence. Guide to the methods of technology appraisal 2013. Published Online First: 2018.https://www.nice.org.uk/process/pmg9/resources/guide-to-the-methods-of-technology-appraisal-2013-pdf-2007975843781

52 Curtis L, Burns A. Unit Costs of Health and Social Care. Kent: 2019. doi:10.22024/UniKent/01.02.79286

53 Braun V, Clarke V. Using thematic analysis in psychology. *Qual Res Psychol* 2006;**3**:77–101.

54 Borland R., Partos TR, Yong HH, Cummings KM, Hyland A. How much unsuccessful quitting activity is going on among adult smokers? Data from the International Tobacco Control Four Country cohort survey. *Addiction* 2012;**107**:673–82. doi:10.1111/j.1360-0443.2011.03685.x

55 Abroms LC, Westmaas JL, Bontemps-Jones J, Ramani R, Mellerson J. A content analysis of popular smartphone apps for smoking cessation. *Am J Prev Med* 2008;**23**:1–7. doi:10.1038/jid.2014.371

56 Vilardaga R, Casellas-Pujol E, McClernon JF, *et al.* Mobile Applications for the Treatment of Tobacco Use and Dependence. *Curr Addict Reports* 2019;**6**:86–97. doi:10.1007/s40429-019-00248-0

57 McKay FH, Wright A, Shill J, *et al.* Using health and well-being apps for behavior

For peer review only - http://bmjopen.bmj.com/site/about/guidelines.xhtml

change: A systematic search and rating of apps. *JMIR mHealth uHealth* 2019;**7**:1–11. doi:10.2196/11926

58 Whittaker R, McRobbie H, Bullen C, *et al.* Mobile phone text messaging and app-based interventions for smoking cessation. *Cochrane Database Syst Rev* 2019;**2019**. doi:10.1002/14651858.CD006611.pub5

References from Tables

[1] Emery J, Coleman T, Sutton S, Cooper S, Leonardi-Bee J, Jones M, Naughton F. Uptake of Tailored Text Message Smoking Cessation Support in Pregnancy When Advertised on the Internet (MiQuit): Observational Study. *J Med Internet Res*. 2018;20(4):e146.

Figure 1 Flow diagram showing study design, measures and sample sizes

For peer review only - http://bmjopen.bmj.com/site/about/guidelines.xhtml

Figure 1 Flow diagram showing study design, measures and sample sizes

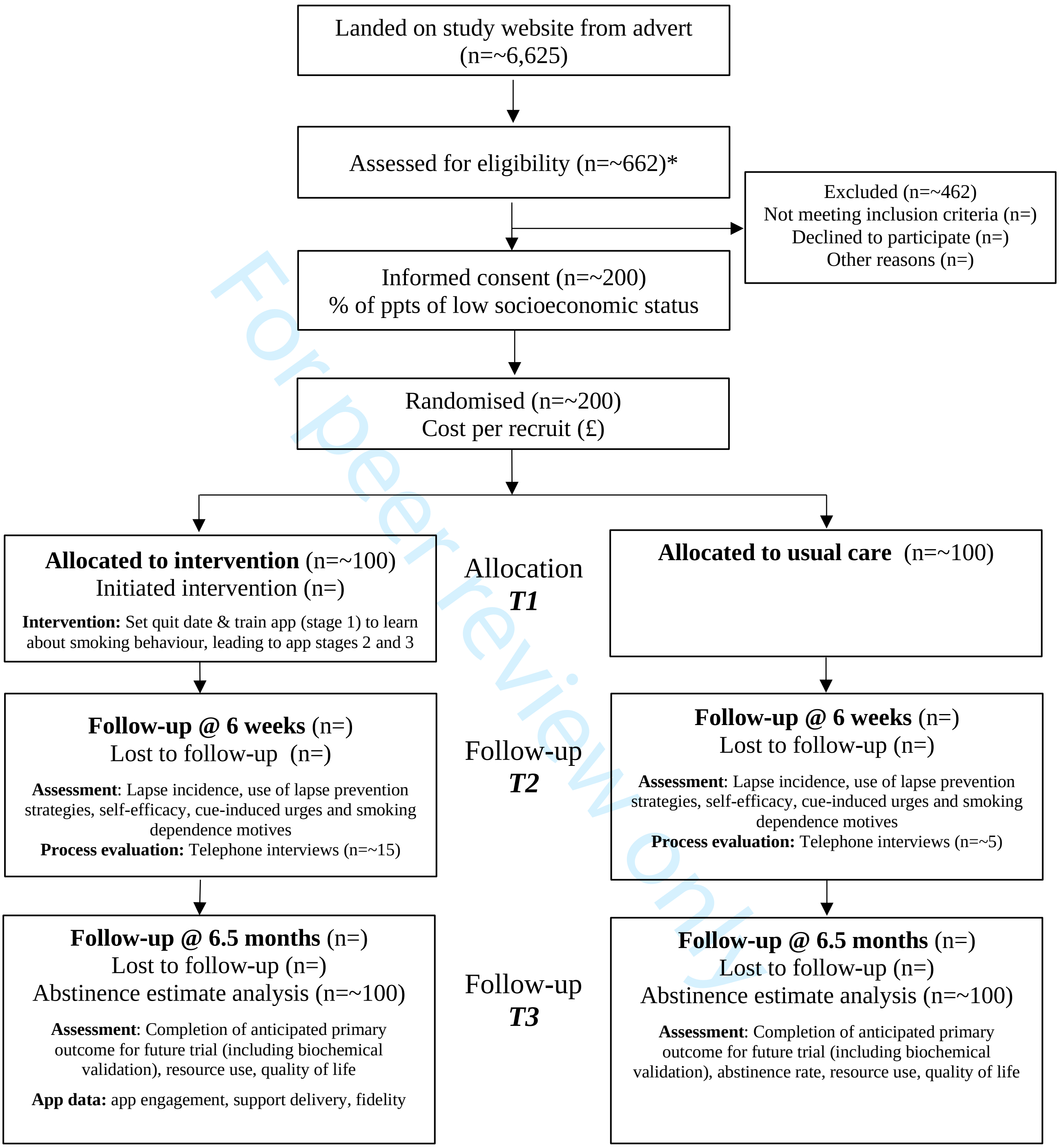

*Based on Emery et al (2018) [1] investigating the online uptake of a text message smoking cessation intervention. Approximately 10% of those visiting the landing page from paid-for Google and Facebook adverts completed eligibility questions, and of those 30% signed up.

For peer review only

Supplementary File

Protocol amendments.

Minor, non-notifiable, August 2020.

Minor, non-notifiable, December 2020.

Amendments to the Protocol and other documents (e.g. changes to eligibility criteria, outcomes, sample size calculations, analyses) will be agreed by the Trial Management Group (TMG). Such amendments will be forwarded to the Sponsor for confirmation as to whether it is either substantial or non-substantial and will then be submitted to the Ethics Committee for categorisation and approval prior to implementation.

Indemnity

University of East Anglia insurance shall apply.

Ancillary and post-trial care

The end of the trial is defined as 6 months following the last follow-up of the last participant randomised, to allow for data entry and data cleaning activities to be completed.

Once participants' involvement in the study has ended, they will no longer receive communications from the study team other than a copy of the findings, if they have requested one. Participants will be sent an SMS to inform them that their participation in the study has ended.

The NHS SmokeFree website support will continue be available after this study ends. Participants in the app group will still be able to access and use the Quit Sense app on their mobile if they wish to do so. If participants continue to use the app, it will continue to collect research data that we may use as part of the study analyses. After the study ends Quit Sense will no longer have technical support and participants will not be able to update or reinstall it. If participants wish, they can uninstall the app following the usual process for removing apps from their phone.

For peer review only - http://bmjopen.bmj.com/site/about/guidelines.xhtml

Data

Monitoring and management

The Chief Investigator has overall responsibility and will oversee study management. The Trial Management Group will be responsible for the running of the trial and will be supported by the Trial Steering Committee. A separate Data Monitoring and Ethics Committee is not considered necessary because the study is deemed to be low risk to participants.

Study data are collected and managed using REDcap (Research Electronic Data Capture). Data provided by trial participants will be entered under the participants PID number onto the central database stored on the servers based at the Norwich Clinical Trials Unit (NCTU). Access to the database will be via unique, individually assigned (i.e. not generic) usernames and passwords, and only accessible to members of the Quit Sense trial team and external regulators if requested. The servers are protected by firewalls and are patched and maintained according to best practice. The physical location of the servers is protected physically and environmentally in accordance with University of East Anglia's General Information Security Policy 3 (GISP3: Physical and environmental security).

The database and associated code have been developed by NCTU Data Management in conjunction with the Quit Sense trial team. The database software provides several features to help maintain data quality, including; maintaining an audit trail, allowing custom validations on all data, allowing users to raise data query requests, and search facilities to identify validation failure/ missing data. After completion of the trial the database will be retained on the servers of NCTU for on-going analysis of secondary outcomes.

Any transcriptions from the telephone interviews or app collected qualitative data will be anonymised. Voice recordings from telephone interviews and app recordings will be deleted after transcription, or if not transcribed then after audio coding for analysis.

Confidentiality and access to data

The investigators agree to archive and/or arrange for secure storage of Quit Sense trial materials and records for 20 years after the close of the trial unless otherwise advised by the NCTU. Personal contact details such as names, addresses and phone numbers will be destroyed 12 months after the end of the trial. Requests for access to trial data will be

For peer review only - http://bmjopen.bmj.com/site/about/guidelines.xhtml

considered, and approved in writing where appropriate, after formal application to the TMG or Trial Steering Committee (TSC). Considerations for approving access are documented in the TMG and TSC Terms of Reference. Location data will be kept for 20 years before being destroyed. This data will not be made available as an open data set outside of Quit Sense collaborations.

Concomitant Care

All participants will have access to treatment as usual for smoking cessation regardless of randomisation into this trial.

Roles and responsibilities

These membership lists are correct at the time of writing:

Protocol contributors

Name	Affiliation	Role
Felix Naughton	UEA	Led on protocol development
Juliet High	Norwich CTU	Contributed to regulatory and CTU related content
Aimie Hope	UEA	Contributed to protocol development
Caitlin Notley	UEA	Contributed to the qualitative process evaluation section
Lee Shepstone	Norwich CTU	Contributed to the statistical analysis section
Garry Barton	Norwich CTU	Contributed to the health economics section
Tim Coleman	University of Nottingham	Detailed comments on draft

Role of trial sponsor and funders

Name	Affiliation	Role
Helen Sutherland	South Norfolk CCG (‘Host’)	Representative of the host organisation
Graham Horne	UEA (‘Sponsor’)	Representative of the sponsor
Sheila Turner	NIHR	Representative of the funder

For peer review only - http://bmjopen.bmj.com/site/about/guidelines.xhtml

Trial Team

Name	Affiliation	Role and responsibilities
Felix Naughton	UEA	Chief investigator
Juliet High	Norwich CTU	Coordination of CTU contribution to project
Aimie Hope	UEA	Senior Research Associate; supporting trial delivery
Chloe Brown	University of Cambridge	Computer scientist leading app optimisation
Claire West	Norwich CTU	Development of study database and web-platform

Trial Management Group

Name	Affiliation	Role and responsibilities
Felix Naughton	UEA	Chief investigator
Juliet High	Norwich CTU	Coordination of CTU contribution to project
Aimie Hope	UEA	Senior Research Associate; supporting trial delivery
Tim Coleman	UEA	Co-applicant; mentoring support for CI
Caitlin Notley	UEA	Co-applicant; qualitative process evaluation advisor
Garry Barton	Norwich CTU	Co-applicant; health economics oversight
Chloe Brown	University of Cambridge	Computer scientist leading app optimisation
Ann Marie Swart	Norwich CTU	CTU oversight
Claire West	Norwich CTU	Development of study database and web-platform
Graham Horne	UEA	Representative of trial sponsor

Trial Steering Committee

Name	Affiliation	Role and responsibilities
Michael Ussher	St Georges/University of Stirling	Independent chair (clinical/behavioural science)
Aleksandra Herbec	UCL	Independent member (behavioural science)

For peer review only - http://bmjopen.bmj.com/site/about/guidelines.xhtml

Simon Coulton	University of Kent	Independent member (statistics/HE)
Qasim Chowdary	Public Health England	Independent member (services/clinical/policy)
Jo Hardy	PPI	Independent member (PPI)
Felix Naughton	UEA	Non-independent member (voting)
Juliet High	Norwich CTU	Observer
Helen Sutherland	South Norfolk CCG	Observer (host organisation)
Graham Horne	UEA	Observer (sponsor)
Aimie Hope	UEA	Observer

For peer review only

For peer review only - http://bmjopen.bmj.com/site/about/guidelines.xhtml

Appendix 2

Quit Sense Consent form

I understand that my participation is voluntary and that I am free to withdraw at any time, without giving any reason, and without my legal rights being affected.	
I understand that should I withdraw then the information collected so far cannot be erased and that this information may still be used in the project analysis.	
I understand that my personal details will be kept confidential.	
I understand that the data collected about me will be anonymised so that I am not identifiable, that study findings may be disseminated (e.g., in publications, academic conferences), and that fully anonymised data may be shared on academic open access platforms to ensure transparency, aid peer review and allow others to make use of important data for re-analysis.	
I understand and agree that I **may** be invited to verify my smoking status at the end of the study by giving a saliva sample by post, and that giving this sample is optional.	
I understand and agree that I **may** be invited to provide feedback on my experiences of this study and the digital support provided in a recorded telephone interview or by leaving a recorded message, and that giving my feedback is optional.	
I understand that if I provide feedback I may be quoted in published academic journals or presentations at public and academic events in the future, but to maintain my confidentiality I will not be named.	
I agree to my postal address being used in this study and consent to it being passed to the Quit Sense team. I understand my postal address will only be used for this study.	
I understand that **if** I am in the quit smoking app group, I will be offered access to a quit smoking app which will use my phone's in-built sensors (e.g. GPS) to record my location.	
I agree to my mobile phone number being used in this study and consent to it being passed to the Quit Sense team in case I will receive study related text messages. I understand my mobile number will only be used for this study.	
I agree to take part in the above study.	✓
Optional<br>Send me the findings.	✓

For peer review only

I agree to my contact details being held on a secure database until the end of the study so that I may receive a copy of<br>the results.
Name of participant<br><br>Date (entered automatically)

For peer review only

Reporting checklist for protocol of a clinical trial.

Based on the SPIRIT guidelines.

Instructions to authors

Complete this checklist by entering the page numbers from your manuscript where readers will find each of the items listed below.

Your article may not currently address all the items on the checklist. Please modify your text to include the missing information. If you are certain that an item does not apply, please write "n/a" and provide a short explanation.

Upload your completed checklist as an extra file when you submit to a journal.

In your methods section, say that you used the SPIRITreporting guidelines, and cite them as:

Chan A-W, Tetzlaff JM, Altman DG, Laupacis A, Gøtzsche PC, Krleža-Jerić K, Hróbjartsson A, Mann H, Dickersin K, Berlin J, Doré C, Parulekar W, Summerskill W, Groves T, Schulz K, Sox H, Rockhold FW, Rennie D, Moher D. SPIRIT 2013 Statement: Defining standard protocol items for clinical trials. Ann Intern Med. 2013;158(3):200-207

		Reporting Item	Page Number
**Administrative information**			
Title	#1	Descriptive title identifying the study design, population, interventions, and, if applicable, trial acronym	1

For peer review only

Trial registration	#2a	Trial identifier and registry name. If not yet registered, name of intended registry	2
Trial registration: data set	#2b	All items from the World Health Organization Trial Registration Data Set	
Protocol version	#3	Date and version identifier	19
Funding	#4	Sources and types of financial, material, and other support	19
Roles and responsibilities: contributorship	#5a	Names, affiliations, and roles of protocol contributors	1, 19
Roles and responsibilities: sponsor contact information	#5b	Name and contact information for the trial sponsor	20
Roles and responsibilities: sponsor and funder	#5c	Role of study sponsor and funders, if any, in study design; collection, management, analysis, and interpretation of data; writing of the report; and the decision to submit the report for publication, including whether they will have ultimate authority over any of these activities	20<br>Appendix 1 p. 3
Roles and responsibilities: committees	#5d	Composition, roles, and responsibilities of the coordinating centre, steering committee, endpoint adjudication committee, data management team, and	Appendix 1 p. 4-5

For peer review only

		other individuals or groups overseeing the trial, if applicable (see Item 21a for data monitoring committee)	
**Introduction**			
Background and rationale	#6a	Description of research question and justification for undertaking the trial, including summary of relevant studies (published and unpublished) examining benefits and harms for each intervention	4-5
Background and rationale: choice of comparators	#6b	Explanation for choice of comparators	9
Objectives	#7	Specific objectives or hypotheses	5
Trial design	#8	Description of trial design including type of trial (eg, parallel group, crossover, factorial, single group), allocation ratio, and framework (eg, superiority, equivalence, non-inferiority, exploratory)	5
**Methods: Participants, interventions, and outcomes**			
Study setting	#9	Description of study settings (eg, community clinic, academic hospital) and list of countries where data	7

For peer review only

		will be collected. Reference to where list of study sites can be obtained	
Eligibility criteria	#10	Inclusion and exclusion criteria for participants. If applicable, eligibility criteria for study centres and individuals who will perform the interventions (eg, surgeons, psychotherapists)	7
Interventions: description	#11a	Interventions for each group with sufficient detail to allow replication, including how and when they will be administered	9-12
Interventions: modifications	#11b	Criteria for discontinuing or modifying allocated interventions for a given trial participant (eg, drug dose change in response to harms, participant request, or improving / worsening disease)	9
Interventions: adherance	#11c	Strategies to improve adherence to intervention protocols, and any procedures for monitoring adherence (eg, drug tablet return; laboratory tests)	9
Interventions: concomitant care	#11d	Relevant concomitant care and interventions that are permitted or prohibited during the trial	Appendix 1 p. 3
Outcomes	#12	Primary, secondary, and other outcomes, including the specific measurement variable (eg, systolic blood pressure), analysis metric (eg, change from baseline, final value, time to event), method of aggregation (eg, median, proportion), and time point for each outcome. Explanation of the clinical relevance of chosen	12-14

For peer review only

		efficacy and harm outcomes is strongly recommended	
Participant timeline	#13	Time schedule of enrolment, interventions (including any run-ins and washouts), assessments, and visits for participants. A schematic diagram is highly recommended (see Figure)	Schedule pp. 6-7 Figure 1
Sample size	#14	Estimated number of participants needed to achieve study objectives and how it was determined, including clinical and statistical assumptions supporting any sample size calculations	14
Recruitment	#15	Strategies for achieving adequate participant enrolment to reach target sample size	8
**Methods: Assignment of interventions (for controlled trials)**			
Allocation: sequence generation	#16a	Method of generating the allocation sequence (eg, computer-generated random numbers), and list of any factors for stratification. To reduce predictability of a random sequence, details of any planned restriction (eg, blocking) should be provided in a separate document that is unavailable to those who enrol participants or assign interventions	14-15

For peer review only

Allocation concealment mechanism	#16b	Mechanism of implementing the allocation sequence (eg, central telephone; sequentially numbered, opaque, sealed envelopes), describing any steps to conceal the sequence until interventions are assigned	15
Allocation: implementation	#16c	Who will generate the allocation sequence, who will enrol participants, and who will assign participants to interventions	15
Blinding (masking)	#17a	Who will be blinded after assignment to interventions (eg, trial participants, care providers, outcome assessors, data analysts), and how	15
Blinding (masking): emergency unblinding	#17b	If blinded, circumstances under which unblinding is permissible, and procedure for revealing a participant's allocated intervention during the trial	N/A
**Methods: Data collection, management, and analysis**			
Data collection plan	#18a	Plans for assessment and collection of outcome, baseline, and other trial data, including any related processes to promote data quality (eg, duplicate measurements, training of assessors) and a description of study instruments (eg, questionnaires, laboratory tests) along with their reliability and	Schedule p. 6-7<br>Procedure p. 8-9

For peer review only

		validity, if known. Reference to where data collection forms can be found, if not in the protocol	Intervention (app) p. 10-12
Data collection plan: retention	#18b	Plans to promote participant retention and complete follow-up, including list of any outcome data to be collected for participants who discontinue or deviate from intervention protocols	9
Data management	#19	Plans for data entry, coding, security, and storage, including any related processes to promote data quality (eg, double data entry; range checks for data values). Reference to where details of data management procedures can be found, if not in the protocol	Appendix 1 p. 2-3
Statistics: outcomes	#20a	Statistical methods for analysing primary and secondary outcomes. Reference to where other details of the statistical analysis plan can be found, if not in the protocol	15-16
Statistics: additional analyses	#20b	Methods for any additional analyses (eg, subgroup and adjusted analyses)	16-17
Statistics: analysis population and missing data	#20c	Definition of analysis population relating to protocol non-adherence (eg, as randomised analysis), and any statistical methods to handle missing data (eg, multiple imputation)	15

For peer review only

Methods: Monitoring

Data monitoring: formal committee	#21a	Composition of data monitoring committee (DMC); summary of its role and reporting structure; statement of whether it is independent from the sponsor and competing interests; and reference to where further details about its charter can be found, if not in the protocol. Alternatively, an explanation of why a DMC is not needed	Appendix 1 p. 2-3
Data monitoring: interim analysis	#21b	Description of any interim analyses and stopping guidelines, including who will have access to these interim results and make the final decision to terminate the trial	N/A
Harms	#22	Plans for collecting, assessing, reporting, and managing solicited and spontaneously reported adverse events and other unintended effects of trial interventions or trial conduct	N/A
Auditing	#23	Frequency and procedures for auditing trial conduct, if any, and whether the process will be independent from investigators and the sponsor	N/A

Ethics and dissemination

Research ethics approval	#24	Plans for seeking research ethics committee / institutional review board (REC / IRB) approval	19

For peer review only

Protocol amendments	#25	Plans for communicating important protocol modifications (eg, changes to eligibility criteria, outcomes, analyses) to relevant parties (eg, investigators, REC / IRBs, trial participants, trial registries, journals, regulators)	Appendix 1 p. 1
Consent or assent	#26a	Who will obtain informed consent or assent from potential trial participants or authorised surrogates, and how (see Item 32)	8 Appendix 2
Consent or assent: ancillary studies	#26b	Additional consent provisions for collection and use of participant data and biological specimens in ancillary studies, if applicable	8
Confidentiality	#27	How personal information about potential and enrolled participants will be collected, shared, and maintained in order to protect confidentiality before, during, and after the trial	Appendix 1 p. 2-3
Declaration of interests	#28	Financial and other competing interests for principal investigators for the overall trial and each study site	19
Data access	#29	Statement of who will have access to the final trial dataset, and disclosure of contractual agreements that limit such access for investigators	Appendix 1 p. 2-3
Ancillary and post trial care	#30	Provisions, if any, for ancillary and post-trial care, and for compensation to those who suffer harm from trial participation	Appendix 1 p. 1

For peer review only

Dissemination policy: trial results	#31a	Plans for investigators and sponsor to communicate trial results to participants, healthcare professionals, the public, and other relevant groups (eg, via publication, reporting in results databases, or other data sharing arrangements), including any publication restrictions	2
Dissemination policy: authorship	#31b	Authorship eligibility guidelines and any intended use of professional writers	N/A
Dissemination policy: reproducible research	#31c	Plans, if any, for granting public access to the full protocol, participant-level dataset, and statistical code	See open data statement
**Appendices**			
Informed consent materials	#32	Model consent form and other related documentation given to participants and authorised surrogates	Appendix 2
Biological specimens	#33	Plans for collection, laboratory evaluation, and storage of biological specimens for genetic or molecular analysis in the current trial and for future use in ancillary studies, if applicable	9,13

None The SPIRIT checklist is distributed under the terms of the Creative Commons Attribution License CC-BY-ND 3.0. This checklist can be completed online using https://www.goodreports.org/, a tool made by the EQUATOR Network in collaboration with Penelope.ai
