# Supplementary material for: Randomised controlled trial of a just-in-time adaptive intervention (JITAI) smoking cessation smartphone app: the Quit Sense feasibility trial protocol
Source: BMJ Open. 2021 Apr 26;11(4):e048204. doi: 10.1136/bmjopen-2020-048204 (PMC8076923; doi:10.1136/bmjopen-2020-048204)
Supplement: Reviewer comments [file bmjopen-2020-048204.reviewer_comments.pdf]

PEER REVIEW HISTORY

BMJ Open publishes all reviews undertaken for accepted manuscripts. Reviewers are asked to complete a checklist review form (**http://bmjopen.bmj.com/site/about/resources/checklist.pdf**) and are provided with free text boxes to elaborate on their assessment. These free text comments are reproduced below.

ARTICLE DETAILS

**TITLE (PROVISIONAL)**	Randomised controlled trial of a Just-In-Time Adaptive Intervention (JITAI) smoking cessation smartphone app: The Quit Sense feasibility trial protocol
**AUTHORS**	Naughton, Felix; Brown, Chloë; High, Juliet; Notley, Caitlin; Mascolo, Cecilia; Coleman, Tim; Barton, Garry; Shepstone, Lee; Sutton, Stephen; Prevost, A.; Crane, David; Greaves, Felix; Hope, Aimie

This article was not externally reviewed at BMJ Open. Protocols that have been independently assessed prior to submission to BMJ Open are usually fast-tracked to publication on the grounds that further substantial changes will not be possible. This independent assessment will usually be external, independent review for both a substantial grant award from a non-commercial or government funder and ethics approval.
